# Identification of a Conserved Non-Protein-Coding Genomic Element that Plays an Essential Role in *Alphabaculovirus* Pathogenesis

**DOI:** 10.1371/journal.pone.0095322

**Published:** 2014-04-16

**Authors:** Irina Kikhno

**Affiliations:** Institute of Molecular Biology & Genetics of Ukrainian Academy of Science, Kiev, Ukraine; Centro Nacional de Biotecnologia (CNB-CSIC), Spain

## Abstract

Highly homologous sequences 154–157 bp in length grouped under the name of “conserved non-protein-coding element” (CNE) were revealed in all of the sequenced genomes of baculoviruses belonging to the genus *Alphabaculovirus*. A CNE alignment led to the detection of a set of highly conserved nucleotide clusters that occupy strictly conserved positions in the CNE sequence. The significant length of the CNE and conservation of both its length and cluster architecture were identified as a combination of characteristics that make this CNE different from known viral non-coding functional sequences. The essential role of the CNE in the *Alphabaculovirus* life cycle was demonstrated through the use of a CNE-knockout *Autographa californica* multiple nucleopolyhedrovirus (AcMNPV) bacmid. It was shown that the essential function of the CNE was not mediated by the presumed expression activities of the protein- and non-protein-coding genes that overlap the AcMNPV CNE. On the basis of the presented data, the AcMNPV CNE was categorized as a complex-structured, polyfunctional genomic element involved in an essential DNA transaction that is associated with an undefined function of the baculovirus genome.

## Introduction

Certain processes governing basic DNA transactions such as replication, transcription, and site-specific recombination appear to be strictly related to precisely located non-coding genomic regions (non-coding functional elements, *nfes*) that specifically interact with the proteins involved in these processes. Numerous *nfes* have been identified in viral genomes, including the common elements shared by all life forms (e.g., transcriptional regulatory sequences, replication origins (*oris*)) and virus-specific elements. The latter involve viral DNA processing and packaging signals, which are characteristic of the vast majority of viruses [1], and signals that govern the integration and excision of viral DNA into and out of the host genome, which are characteristic of some integrative phages [2] and viruses that are exemplified by adeno-associated viruses [3], retroviruses [4], and herpesviruses [5]. The diversity of strategies developed by different viruses to successfully propagate themselves has resulted in a wide variety of viral *nfes* with regard to structure and mechanism of action.

The family Baculoviridae comprises a diverse collection of arthropod-specific viruses with large, covalently closed, circular double-stranded DNA genomes that are predicted to comprise up to 180 genes [6]. The family is traditionally divided into two genera, namely *Nucleopolyhedrovirus* (NPV) and *Granulovirus* (GV), on the basis of the morphology of the occlusion bodies (polyhedra and granules, respectively) produced by the members of each genus in the final stage of infection. Modern taxonomy reflects the co-evolution of baculoviruses with their hosts, and accordingly, the Baculoviridae family includes the *Alphabaculovirus, Betabaculovirus*, *Deltabaculovirus*, and *Gammabaculovirus* genera represented by lepidopteran NPVs (classified into Group I and II), lepidopteran GVs, dipteran NPVs, and hymenopteran NPVs, respectively [6]. A comparative analysis of baculoviral genomes has revealed that baculoviruses are highly diverse in terms of their GC content, genome length, gene content, and gene organization [7] [8] [9], which likely reflects their long evolutionary history in adapting to different hosts. The conservation of the general mechanisms of baculoviral pathogenesis can be deduced from comparative genomic studies; specifically, 37 core genes shared by all sequenced baculovirus genomes [10] [11] and common DNA *nfes* have been identified. The latter include (i) homologous repeat regions (*hrs*), specifically organized sequences interspersed at multiple locations throughout the genomes of representatives of all baculovirus genera [10], which function as enhancers [12] [13] and *oris* [14] [15], and (ii) the so-called non-*hr oris* revealed in both *Alphabaculovirus* [16] [17] [18] [19] and *Betabaculovirus* [20] genomes.

Although baculoviruses comprise a well-characterized family, the molecular mechanisms of some basic aspects of their genome function, such as replication, packaging into a capsid, and transition to the latent state, are not yet well understood [10] [21] [22]. By analogy with other viruses, all of these processes are expected to be associated with some specialized *nfes* that may be hidden within the context of the large and complex baculovirus genome.

The strategy for the identification of baculovirus *nfes* implemented by the present study was determined by a previous observation in *Malacosoma neustria* (Mane) NPV propagated in an *Antheraea pernyi* (Anpe) ovarian cell culture. The infection process characteristic of this virus-cell system was associated with the emergence of linear viral genomes, a phenomenon that has never been reported before in relation to other baculovirus-host systems. The “break site”, referred to herein as the “linearization site”, was localized to a defined locus on the ManeNPV genome map [23]. The terminal sequences of the linear ManeNPV genome were considered to be of great interest because, when linked together in a circular genome, the terminal sequences were expected to compose an *nfe* involved in some unexplored aspect of baculoviral genome activity that is associated with the conversion of the original circular DNA into a linear derivative.

In the present study, the ManeNPV genome fragment comprising the linearization site was cloned, sequenced, and subjected to a BLAST homology search. The ManeNPV 155-bp sequence containing the linearization site was shown to exhibit a high degree of homology with 154–157-bp sequences found in all *Alphabaculovirus* genomes sequenced to date. A comparative sequence analysis demonstrated that, although the homologous sequences overlap with short, putative protein-coding ORFs in the genomes of some viruses, the non-protein-coding content of the sequence is likely to be subject to strong negative selection. This observation became the basis for categorizing the newly identified element as a conserved non-protein-coding element (CNE). An examination of the consensus sequence obtained by aligning the CNEs extracted from 38 *Alphabaculovirus* genomes revealed highly conserved nucleotide clusters represented by specifically arranged repeated sequences. Based on the data from the comparative analysis, the experimental data obtained using a CNE-deficient AcMNPV bacmid, and the relevant literature data the CNE was postulated to be a polyfunctional genomic element involved in key process(es) of *Alphabaculovirus* pathogenesis.

## Results

### Detection of the conserved element in *Alphabaculovirus* genomes

**Figure 1:**
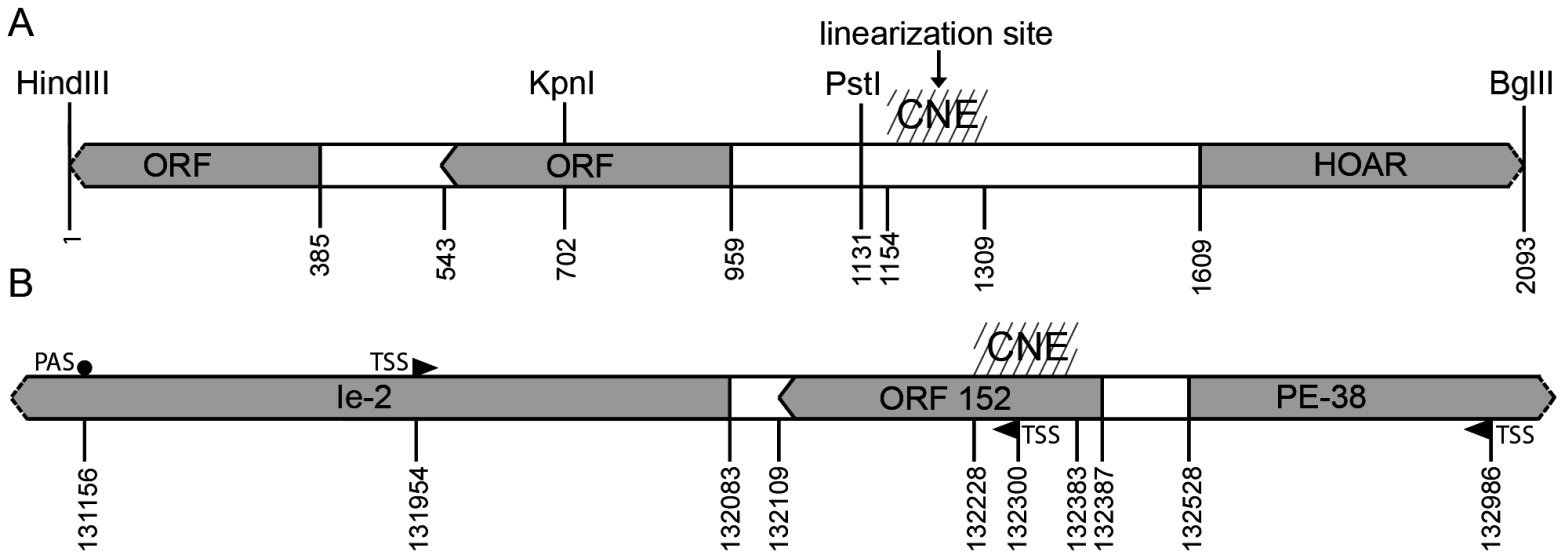
Schematic representation of the viral genome fragments enclosing the CNE. **A**. A BamHI fragment of the ManeMNPV genome (GenBank accession number for the ManeNPV genomic fragment sequence is KF460031). The location of the CNE, the linearization site, the *hoar* gene, and unidentified ORFs are indicated. **B**. A fragment of the AcMNPV genome (GenBank accession number for the complete AcMNPV genome sequence is NC_001623). The location of the CNE, the OFR152, the *ie2* and *pe38* genes are indicated. Nucleotide positions correspond to sequences deposited at GenBank.

The linearization site was previously delineated on the physical map of the ManeNPV genome with respect to the *Bam*HI, *Kpn*I, and *Pst*I cleavage sites [23]. In the present study, the *Bam*HI-D fragment of the genome harboring the linearization site was cloned into pBR325. A physical map indicating the *Kpn*I, *Bam*HI, *Pst*I, *Bgl*II, and *Hind*III cleavage sites of the resulting recombinant plasmid pMB-D was constructed (Figure S1), and the linearization site was positioned on the plasmid map less than 100 bp downstream of the *Pst*I site, in accordance with its location on the physical map of the ManeNPV genome. The smallest fragment containing the linearization site, a 2.1-kbp *Bgl*II-*Hind*III fragment, was subsequently subcloned into pUC18 and sequenced (GenBank accession number KF460031). Three ORFs longer than the usual gene length limit of 150 bp were detected within the nucleotide sequence of the fragment (Figure 1A) (Figure S2). A BLAST search across the nucleotide databases revealed homology between one of three ORFs and baculovirus *hoar*, a gene encoding a zinc-finger-containing protein with an as-yet-undefined function [24]. In addition, numerous sequences homologous to the nucleotide segment representing the central part of the 649-bp spacer region between *hoar* and the ORF were identified. These sequences were detected in all *Alphabaculovirus* genomes sequenced to date, whereas a BLAST search failed to detect such a conserved element (CE) in the rest of the baculovirus genomes available in the databases, namely in 14 *Betabaculovirus*, 3 *Gammabaculovirus*, and 1 *Deltabaculovirus* genomes. To determine the CE boundaries, CE-containing regions of approximately 200 bp in length from 38 *Alphabaculovirus* genome sequences were aligned. The CEs represented by the conserved portions of the compared sequences were found to range from 154 to 157 bp in length. The CE alignment is shown in Figure S3, and the list of alphabaculoviruses and the CE location in the genome of each virus are shown in Table 1; the abbreviations for the virus names used herein are also provided in this table. The recently published baculovirus phylogenetic tree was used as a source of information regarding the evolutionary relationships between the viruses for which CEs were compared [9]. The ClustalW alignment summary (Table S1) was analyzed to estimate the degree of CE sequence conservation between 38 *Alphabaculovirus* species, revealing a high degree of CE sequence similarity, ranging from 66% (for a pair of distantly related viruses, such as EupsNPV-OpMNPV) to 100% (for some virus pairs that appear to be variants of each other, e.g., BomaNPV-BmNPV).

**Table 1:** The location of the conserved element and the ORFs that overlap this element in *Alphabaculovirus* genomes.

N	Virus	Reference	Accession	Conserved element location	ORF location
1	*Adoxophyes honmai* NPV (AdhoNPV)	[25]	NC_004690	8359–8514	-
2	*Adoxophyes orana* NPV (AdorNPV)	[26]	NC_011423	8164–8319	-
3	*Agrotis ipsilon* MNPV (AgipMNPV)	-	NC_011345	6150–6304	(5964-6200)
4	*Agrotis segetum* NPV (AgseNPV)	[27]	NC_007921	5925–6078	-
5	*Antheraea pernyi* MNPV (AnpeMNPV)	[28]	NC_008035	120238–120393	-
6	*Anticarsia gemmatalis* MNPV (AgMNPV)	[29]	NC_008520	(126126–126282)	-
7	*Apocheima cinerarium* NPV (ApciNPV)	-	NC_018504	(118630–118795)	-
8	*Autographa californica* MNPV (AcMNPV)	[30]	NC_001623	132228–132383	(132109–132387)
*9*	*Bombyx mandarina* NPV (BomaNPV)	[31]	NC_012672	121307–121462	-
10	*Bombyx mori* NPV (BmNPV)	[32]	NC_001962	122075–122230	-
11	*Choristoneura fumiferana* MNPV (CfMNPV)	[33]	NC_004778	124720–124874	-
12	*Choristoneura fumiferana* defective NPV (CfDEFNPV)	[34]	NC_005137	(125538–125694)	125536–125706
13	*Chrysodeixis chalcites* NPV (ChchNPV)	[35]	NC_007151	6659–6813	-
14	*Clanis bilineata* NPV (ClbiNPV)	[36]	NC_008293	(6290–6444)	-
15	*Ecotropis obliqua* NPV (EcobNPV)	[37]	NC_008586	6489–66444	-
16	*Epiphyas postvittana* NPV (EppoNPV)	[38]	NC_003083	114636–114791	-
17	*Euproctis pseudoconspersa* NPV (EupsNPV)	[39]	NC_012639	5241–5395	5295–5465
18	*Helicoverpa armigera* MNPV (HearMNPV)	-	NC_011615	9236–9390	-
19	*Helicoverpa armigera* SNPV (HearSNPV)	[40]	NC_003094	5422–5576	(5447–5638)
20	*Hyphantria cunea* NPV (HycuNPV)	[41]	NC_007767	7138–7293	7249–7434
21	*Leucania separata* NPV (LeseNPV)	[42]	NC_008348	8486–8641	-
22	*Lymantria dispar* MNPV (LdMNPV)	[43]	NC_001973	10032–10185	(10115–10474)
23	*Lymantria xylina* MNPV (LyxyMNPV)	[44]	NC_013953	8250–8383	-
24	*Malacosoma neustria* MNPV (ManeMNPV)	-	KF460031	1154–1308	-
25	*Mamestra brassicae* MNPV (MbMNPV)	-	JQ_798165	(9208–9362)	-
26	*Mamestra configurata* NPV-A (MacoNPV-A)	[45]	NC_003529	(9030–9184)	9003–9206
27	*Mamestra configurata* NPV-B (MacoNPV-B)	[46]	NC_004117	(9209–9363)	-
28	*Maruca vitrata* MNPV (MaviMNPV)	[47]	NC_008725	107517–107672	(107491–107676)
29	*Orgyia leucostigma* NPV (Orle NPV)	-	NC_010276	(5410–5565)	-
30	*Orgyia pseudotsugata* MNPV (OpMNPV)	[48]	NC_001875	(128282–128437)	128186–128416
31	*Plutella xylostella* MNPV (PlxyMNPV)	[49]	NC_008349	132750–132905	(132625–132909)
32	*Rachiplusia ou* MNPV (RoMNPV)	[50]	NC_004323	129845–13000	(129726–130041)
33	*Spodoptera exigua* MNPV (SeMNPV)	[51]	NC_002169	5872–6026	-
34	*Spodoptera frugiperda* MNPV (SfMNPV)	[52]	NC_009011	5084–5238	5124–6578
35	*Spodoptera litura* NPVII (SpliNPVII)	-	NC_011616	5412–5567	-
36	*Spodoptera litura* MNPV (SpliMNPV)	[53]	NC_003102	6030–6183	-
37	*Thysanoplusia orichalcea* MNPV (ThorMNPV)	[54]	JX467702	130810–130971	(130802–130975)
38	*Trichoplusia ni* SNPV (TniSNPV)	[55]	NC_007383	5859–6015	-

### The CE encompasses the linearization site of the ManeNPV genome

An examination of the nucleotide sequence of the ManeNPV *Bgl*II-*Hind*III fragment (Figure S2) revealed that the *Pst*I site, which is located proximal to the linearization site on the ManeNPV physical map [23], is situated 17 bp upstream of the CE. Based on the comparison of the *Pst*I site position (17 bp upstream of the CE, in accordance with the sequencing data, and less than 100 bp upstream of the linearization site, in accordance with the ManeNPV physical map, with the CE size (155 bp), it was concluded that the linearization site must be located within the CE. Thus, the CE may be a genomic element involved in the transition from the linear to the circular form of the ManeNPV genome.

### The CE is a non-protein-coding element

Seven ORFs comprising more than 150 nucleotides were shared exclusively by the *Alphabaculovirus* genomes [9]. An analysis of their localizations demonstrated that none of them overlapped with the CE region, and, therefore, the possibility that the CE encodes a conserved alphabaculovirus-specific protein was excluded. However, although ORFs with more than 50 codons are more commonly considered potential protein-coding regions, shorter ORFs should not be absolutely ignored; in fact, they may encode functional, short oligopeptides. To test the potential of CEs of different alphabaculoviruses to encode common short oligopeptide, an examination of the ORFs that overlap each CE locus was performed. A BLAST-based comparative analysis of the amino acid sequences of the polypeptides potentially encoded by numerous ORFs that completely or partially overlap the CEs or their complements failed to identify a putative product shared by all alphabaculoviruses. Thus, it was concluded that a putatively functional, conserved element of *Alphabaculovirus* genomes may be represented by the non-protein/oligopeptide-coding sequence. The above conclusion does not, however, preclude the possibility that the CE could overlap with protein-coding sequences in the genomes of certain *Alphabaculovirus* species. Thirteen of the ORFs that overlapped the CE in *Alphabaculovirus* genomes were selected as the most probable protein gene candidates (Table 1), as their lengths were found to be more than 150 bp.

The term “conserved non-protein-coding element” (CNE) was used to name the newly identified sequence.

### The CNE is characterized by a high AT content

As a first approach to perform a CNE analysis, the AT content of each CNE was compared with the AT content of the resident genome. The analysis revealed that, while the AT content of an alphabaculovirus genome varied between 42.5% (LdMNPV) and 66.6% (ApciNPV), the AT content of the CNE ranged from 55% (LdMNPV) to 73.9% (ClbiNPV) (Table S2). The average AT content of the CNE was 62.6%, quite higher than the genome average of 57.4%. As each CNE subjected to the analysis was found to have higher percentage of AT than the corresponding genome, and as the AT content of each CNE was found to exceed 50%, the CNE was categorized as an AT-rich genomic region.

### The general CNE architecture is highly conserved

The localization of the CNE in the AcMNPV genome made it possible to relate this element to three previously characterized short regions of the AcMNPV genome. A DNase footprinting assay of the promoter-containing region of AcMNPV *pe38* conducted by Krappa and coworkers [56] resulted in the detection of three DNA loci that were protected from DNase digestion located upstream of the *pe38* promoter within the sequence currently recognized as the CNE. Two of these loci were shown to be coupled to some protein(s) both in uninfected cells and in AcMNPV-infected cells at 3 hours post-infection (h p.i.). These loci were represented by the near-identical 33-bp regions of symmetry (132221–132253, 132358–132390) (Figure S4). The third sequence, positioned in a 27-bp region of symmetry (132289–132315), was occupied by protein(s) at 40 h p.i.

**Figure 2:**
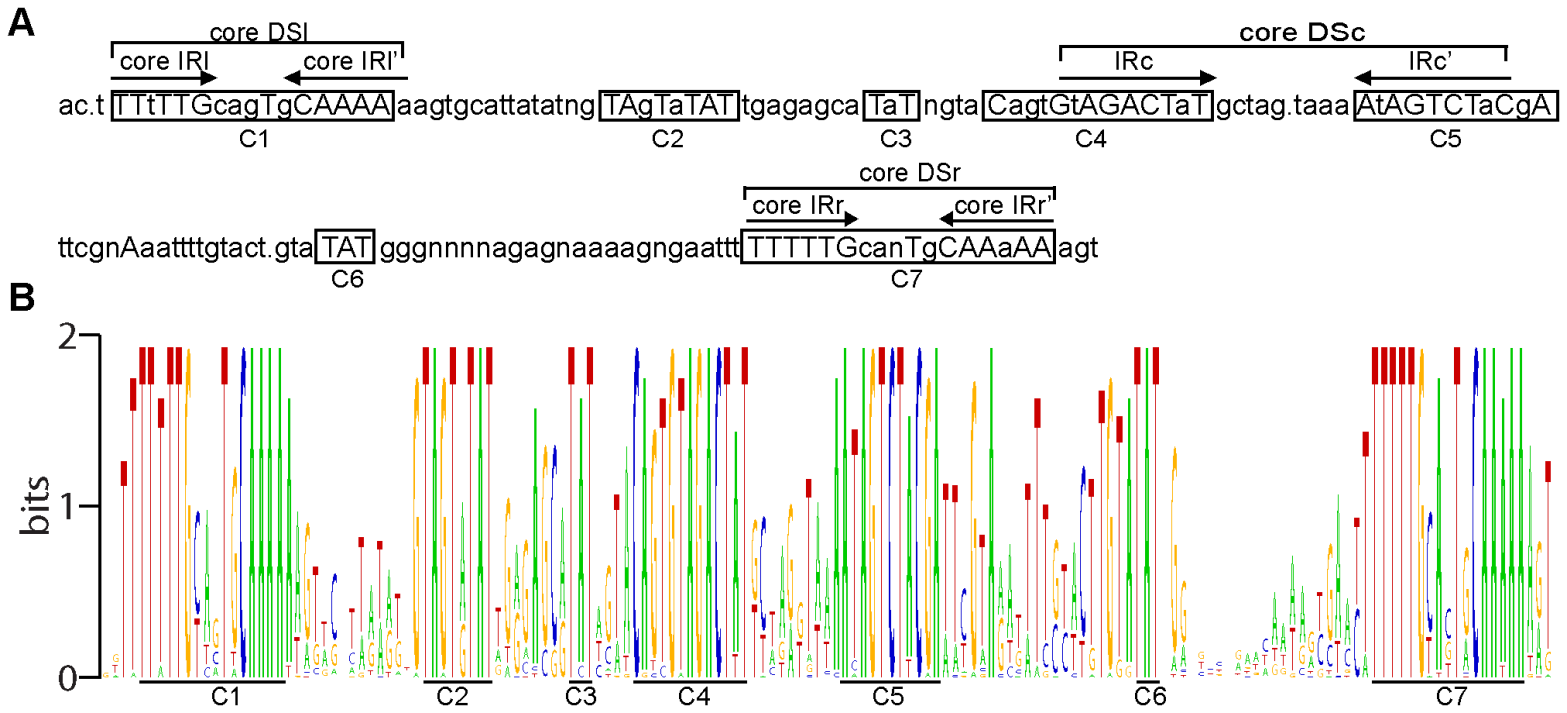
The profiles of CNE sequence conservation. **A**. The consensus sequence determined by comparing the aligned CNEs of 38 alphabaculoviruses. The uppercase letters denote the completely conserved nucleotides, and the lowercase letters denote nucleotides with 50% or greater conservation. The letter “n” indicates that no completely or highly conserved nucleotides were found at the corresponding position. The points mark the positions in which the majority of the aligned sequences contain a gap. The clusters of conserved nucleotides are boxed, and each cluster is marked by the letter c, followed by a number. The lines mark dyad symmetry elements, each of which is indicated by the abbreviation DS in conjunction with the lowercase letters (l, c, r) that specify the DS position in the CNE (left, central, right, respectively). The inverted repeats are indicated with arrows, and the abbreviation IR in conjunction with the letters l, c, and r, assigns each IR pair to a particular DS. **B**. WebLogo for the ClustalW alignment of the CNEs. The CNE consensus sequence is represented graphically as stacks of letters, one stack for each position in the alignment: G is orange; T is red; C is blue; and A is green. The heights of the letters are a measure of nucleotide conservation. Two bits correspond to 100% conservation. The conserved clusters corresponding to those in the consensus sequence are underlined and marked.

The high degree of evolutionary conservation of the particular nucleotides/amino acids and their relative positions across nucleic acid/protein sequence can be indicative of the structural and/or functional significance of the above-named structural units of biopolymers. Thus, to identify functionally/structurally significant nucleotides in the CNE and to determine whether the protein-binding sites found in the AcMNPV CNE are sufficiently conserved across alphabaculoviruses to be considered as elements associated with the conserved function of the CNE, a consensus sequence was determined by comparing 38 aligned CNEs (Figure 2A). The WebLogo tool [57] was used to display the sequence conservation between the 38 CNEs (Figure 2B).

The consensus sequence scanning revealed 50 absolutely conserved bases, which formed 7 discrete clusters along the entire CNE length. An uneven distribution of conserved nucleotides along the CNE length was clearly observed in the CNE sequence logo, a graphical representation of a CNE multiple sequence alignment (Figure 2B). Interestingly, as AT pairs represent 76% of the absolutely conserved bases of the CNE sequence, they constitute a remarkable proportion of the sequences of the conserved clusters. The discontinuous pattern of nucleotide conservation within the CNE, as characterized by the presence of highly variable and highly conserved regions, strongly suggests that highly conserved regions of the CNE are functionally/structurally significant regions under strong negative selection rather than background conservation.

Seven conserved nucleotide clusters found in the CNE were divided into two categories, dyad symmetry elements (DSs) and TAT-containing sequences, according to their structure and nucleotide composition. Two nucleotide clusters (cluster 1 and cluster 7) localized at the CNE termini were found to be represented by similar imperfect DSs (named the left DS (DSl) and right DS (DSr), respectively), each of which was organized as a pair of short A/T-rich inverted repeats (IRs) separated by five asymmetric nucleotides. A comparative analysis of all the DSls and all the DSrs found in the 38 CNEs (Figure S5) demonstrated that a minimal DS arm shared by the vast majority of the DSs analyzed was represented by the hexanucleotide TTTTTG in the forward and reverse orientations. The arm length of many DSs was found to exceed the 6-bp limit due to the presence of less conserved symmetrical complementary bases located proximally to the 6-bp IR, termed the “core IR”; therefore, the arm length of 71 of the 76 DSs was found to range from 6 to 14 bp. The core IRs different from TTTTTG/CAAAAA were only detected in the DSs of four virus species; specifically, symmetrical T-A/A-T substitutions in IRl and IRr' of AgMNPV and CfDEFNPV (viruses that occupy neighboring branches on the phylogenetic tree) resulted in the transformation of minimal IRs into TTATTG and CAATAA, respectively, and point mutations in both hexameric IRls' and IRr' of ManeMNPV and in a hexameric IRl' of ApMNPV converted them to CAAAAT (Figure S5). Taking into account these five degenerate hexanucleotides, it could be postulated that the WTTWTG motif in the forward and reverse orientations is a common core structural unit of both the DSl and DSr. A minimal 17-bp DS composed of core IRs was designated the “core DS”. It is remarkable, that symmetrical complementary bases framing core DSs in the majority of the CNEs are predominantly represented by T/A (Figure S5), i.e. both DSl and DSr exhibit a tendency to the AT-richness preservation. Further analyses concerning the core Dsl and DSr revealed that these elements are enclosed in the two 33-bp protein-binding regions identified by Krappa and co-workers in the AcMNPV genome (Figure S4).

An additional level of CNE complexity was found to be caused by stretches of conserved nucleotides embedded in cluster 4 and cluster 5 and organized in the form of the DS element (named a central DS (DSc) based on its location). In most of the virus genomes, two nonameric IRs separated by 9–10 non-symmetrical nucleotides, GWAGACTWT and its reverse complement AHAGTCTWC, were recognized as common core structural units of the DSc. DSs with arms reaching 10–11 bp in length were also found in many genomes (Figure S5). A 27-bp core DSc corresponded to a third 27-bp protein-binding region previously localized in the AcMNPV genome by Krappa and co-workers [56].

Further inspection of the conserved nucleotide clusters revealed that five of them included a TAT triplet: the 3′ ends of clusters 2 and 5 and the reverse complement of the 3′ end of cluster 4 were represented by a TAT triplet, whereas clusters 2 and 6 were the TAT triplet itself (Figure 2). It is remarkable that the TAT-containing clusters 4 and 5 also fall into the category of IRs, the arms of DSc. TAT was found in clusters 2 and 6 of all 38 CNEs and in clusters 3, 4, and 5 of the vast majority of CNEs.

Thus, all three DSs and the TAT-containing clusters were found to be conserved between the CNEs analyzed and may be considered to be CNE structural elements associated with the conserved CNE function. Together with the data from Krappa and co-workers, these data suggest that DSs may represent conserved motifs for the binding of trans-acting CNE partners. The putative structural/functional significance of the three TAT-containing clusters remains to be determined.

It is worth noting that the CNE alignment (Figure S3) and the consensus sequence derived from it (Figure 2) demonstrate that the positions of the CNE structural elements relative to each other are highly conserved; specifically, the range of variation in the distances between the clusters is very narrow (1–3 bp). Thus, the CNE primary structure, its elemental composition, and its general architecture are likely subjected to strong selective pressure.

### The AcMNPV genome is the most appropriate model to investigate the role of the CNE in viral infections

AcMNPV was chosen as a target for genetic manipulations aimed at examining the role of the CNE in the infection process because it is a well-studied prototype member of the *Alphabaculovirus* genus. Furthermore, its genome is available in a bacmid form that is designed for the quick generation of recombinant virus genomes in *Escherichia coli*. More importantly, the identification and localization of the CNE in the AcMNPV genome enabled its recognition within the context of previously characterized AcMNPV genomic elements, which may be taken into consideration when determining the CNE role. Among these elements are an upstream activating region of the *ie-2* gene [58], ORF152 (*Ac152*) [59], a polyadenylation signal and three transcription start sites required for the initiation of synthesis of overlapping RNAs in AcMNPV-infected Tni cells [60], and binding sites for proteins [56], [61].

An upstream activating region. An examination of the AcMNPV genome demonstrated that the CNE constitutes the central part of a symmetrically arranged region located between the coding sequences of the divergently transcribed immediate early genes *ie2* and *pe38* [62] (Figure 1B) (Figure S4). Both ie2 and pe38 are transcriptional transactivators, participating in many processes, among which DNA replication (ie2 and pe38) and cell cycle arrest (ie2) [22]. Gene knockout experiments clearly demonstrated a non essential role of pe38 in the AcMNPV infection in *Heliothis virescens* larvae [63]. Although the mutational analysis of the ie2 gene provided evidence of a non essential role of several ie2 activities [64] [65] in virus propagation in Sf21 cells, the dispensability of full ie2 in the AcMNPV life cycle remains questionable. Functional studies demonstrated that the 43-bp sequence (132212–132254) that overlaps DSl, the 43-bp sequence (132345–132387) that overlaps DSr, and a 243-bp (132173–132415) sequence that overlaps the full CNE increased *ie2* core promoter activity [58], but not *pe38* promoter activity when the DSr-containing part of the AcMNPV CNE was tested [56]. Remarkably, the AcMNPV DNA motifs that bind proteins in uninfected and infected cells at early stages of infection (see previous section) are enclosed in 43-bp *ie2* promoter-activating sequences. Thus, according to its location and activity, the AcMNPV CNE can be categorized as an upstream, early transcriptional regulator.

*Ac152*. The AcMNPV CNE was found to overlap completely with the 279-bp ORF *Ac152* (Figure 1B) (Figure S4). *Ac152* is likely to have coding capacity because this ORF, in conjunction with several AcMNPV genes, was shown to mediate nuclear G-actin accumulation (a phenomenon characteristic of baculovirus infection) in transient transfection experiments [59].

Transcripts. A comprehensive analysis of the AcMNPV transcriptome [60] revealed three transcription start sites (TSSs) associated with the synthesis of RNAs that overlap the CNE/*Ac152*-containing region (Figure 1B) (Figure S4). The identified TSSs were located at positions 131594, 132300, and 132968 in the AcMNPV genome. While TSS^132968^ and TSS^132300^ are marks of same-strand overlapping genes (or alternatively, a single gene encoding two overlapping transcripts), TSS^131594^ is assigned to the third overlapping gene that is transcribed from the opposite strand. An analysis of each TSS location relative to *Ac152* suggests that TSS^132968^ is a component of the putative protein-coding gene responsible for the synthesis of *Ac152* product. The detection of six additional upstream start codons in the 381-bp putative 5′-untranslated region of RNA initiated at TSS^132968^ suggests that Ac152 translation from this RNA is rather questionable. However, Ac152 protein expression is not impossible; the translational activity of RNAs of some eukaryotic and viral proteins are regulated by one or several additional AUG located upstream of the true start codon [66] [67]. Furthermore, it has been suggested that different TSSs may be used for the initiation of mRNA during virus propagation in different hosts [60]. Therefore, as TSS^132968^ was predicted on the basis of the AcMNPV transcriptome analysis derived from infected Tni cells, the utilization of an alternative TSS for the expression of Ac152 protein in Sf9 cells cannot be ruled out.

Two other TSSs, TSS^132300^ and TSS^131594^, appear to be the components of likely non-protein-coding RNA (ncRNA) genes, i.e., genes that encode RNA sequences that function without being translated to a protein, as no ORFs longer than 150 bp were found in the 3′- proximal regions of those TSSs.

Target sites for proteins. Three symmetrical protein-binding sites spanning DSl, DSc and DSr of AcMNPV CNE were revealed by Krappa and coworkers [56] (see previous section). Taken together with the above-mentioned studies of Carson and coworkers [58], these data suggest that DSl and DSr of the AcMNPV CNE bind some protein(s) putatively involved in the positive regulation of *ie2* activity. Also, *ie2* expression was shown to be regulated negatively by the viral immediate early protein ie1 through mediation of the ie1 binding site located 82 nucleotides upstream of the CNE [61] (Figure S4). The second ie1 binding site, the mediator of the *pe38* negative regulation was identified 88 nucleotides downstream of the CNE. In addition, the binding site for cellular proteins members of the GATA family was revealed 38 nucleotides downstream of the CNE [56] (Figure S4).

The numerous non-systematized findings concerning the CNE-containing genomic locus of AcMNPV indicate that additional studies are required for a reliable conclusion as to whether sequence conservation of the CNE reflects its conservation as significant part of the ncRNA genes or/and as an important transcriptional cis-regulatory sequence or/and as some additional undefined key *nfe*. The structural analysis of the CNE-containing region became the basis for the design of experiments aimed at investigating the role of each CNE-related functional element in AcMNPV infectivity as well as a guideline to interpret the experimental results.

### Construction of the CNE-disrupted, CNE-repaired and positive control AcMNPV bacmids

**Figure 3:**
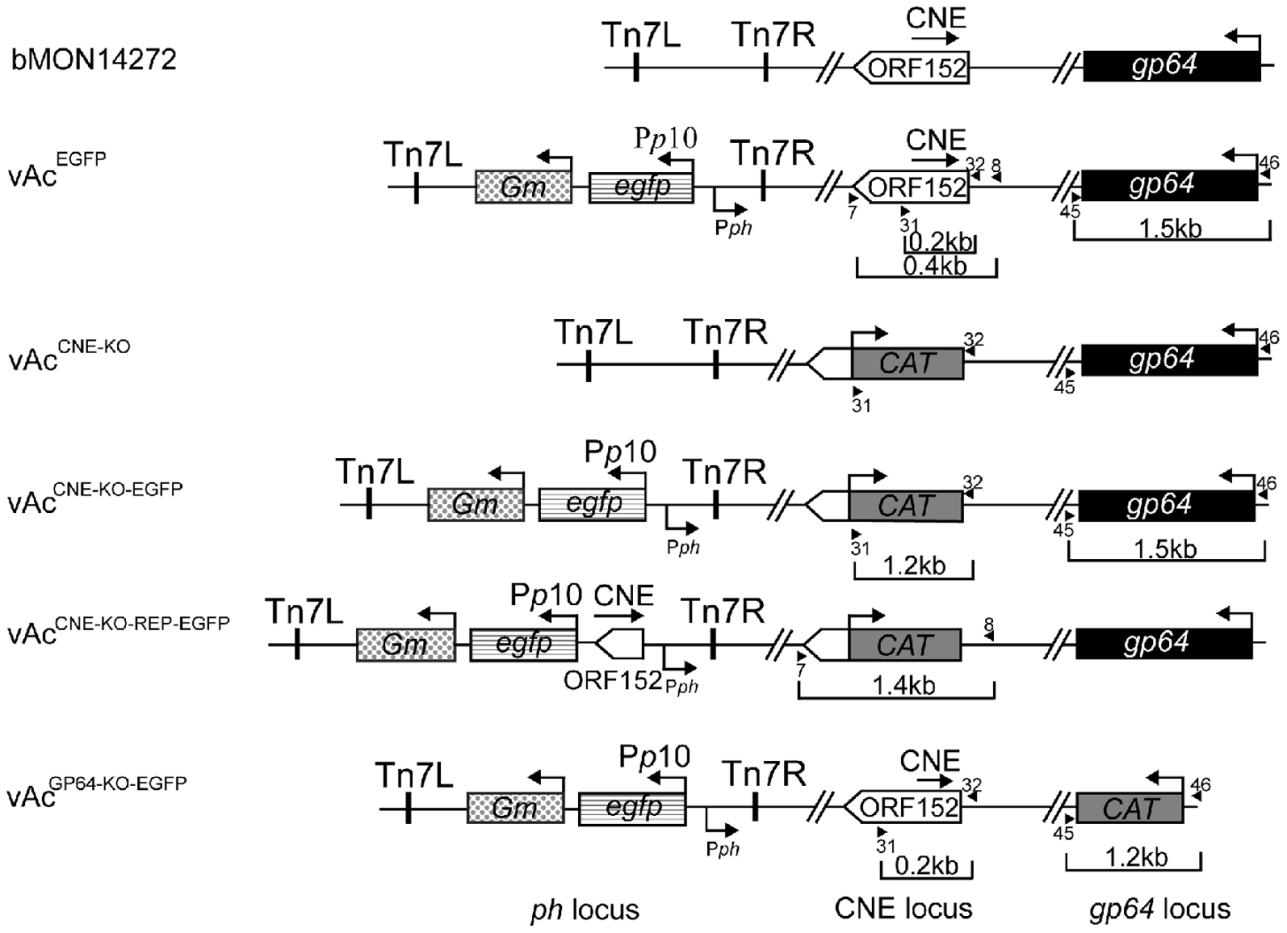
A comparative presentation of the organization of the genome region of the parental bacmid and the organization of the same regions of the recombinant genomes. The recombinant genomes were derived from the *Escherichia coli* system for bacmid bMON14272. An egfp-producing virus (vAc^EGFP^) resulted from the insertion of a gentamicin-resistant gene (*Gm*)-enhanced green fluorescent protein gene (*egfp*) cassette into the *polyhedrin (ph)* locus of bMON14272 via transposon-mediated recombination using the Bac-to-Bac system (Invitrogen Life Technologies). The egfp-expressing CNE- and *gp64*-knockout bacmids, vAc^CNE-KO-EGFP^ and vAc^GP64-KO-EGFP^, respectively, were constructed by replacing the CNE or *gp64* in vAc^EGFP^ with a chloramphenicol acetyltransferase gene (*CAT*) PCR cassette using the λ Red recombination system (see Methods for the PCR primers used). The same technique was used to generate a CNE-knockout virus lacking *egfp* (vAc^CNE-KO^) from bMON14272. vAc^CNE-KO^ was used as an intermediate construct for the generation of the egfp-expressing CNE-repair virus (vAC^CNE-KO-EGFP-REP^) by the site-specific transposition of the *Gm-egfp-CNE* cassette into the *ph* locus using the Bac-to-Bac system. Tn7L and Tn7R indicate the left and right transposon arms, respectively. The arrows, labeled P*p10* and P*ph*, denote the *ph* and *p10* promoters, respectively, and the unlabeled arrows denote native gene promoters. The arrowheads labeled with numbers specify the annealing sites of the respective primers. The boxes labeled by gene names represent the corresponding genes. Different types of shading were used to represent each gene in a visually appealing form. The unshaded arrow-shaped boxes indicate the complete and truncated ORF152, and the black arrow above the boxes specifies the CNE location within ORF152.

Genetic knockouts, gene additions, and gene replacements were used to study the role of the CNE in AcMNPV infection. Four recombinant bacmids were generated (Figure 3). For brevity, the term “CNE” is used herein for the genomic locus targeted by the genetic manipulations; however, *Ac152* enclosed in the CNE, as well as the transcribed sequences that overlap the CNE, are also affected by these manipulations.

The gene encoding enhanced green fluorescent protein (egfp), a reporter of viral infection, was incorporated into the bacmid under the control of the late *p10* promoter in the AcMNPV bacmid bMON14272 using transposon-mediated recombination in *Escherichia coli* cells, as previously described [68]. The resulting bacmid vAc^EGFP^ served as a positive control and an intermediate construct for the generation of the egfp-expressing CNE-knockout bacmid.

vAc^CNE-KO-EGFP^, an *egfp*-containing CNE-knockout bacmid, was generated by the replacement of the CNE in the vAc^EGFP^ genome with a chloramphenicol acetyltransferase (CAT) gene in *Escherichia coli* cells using the λ Red recombination system.

vAc^CNE-KO-REP-EGFP^, the CNE-repair bacmid, was generated in two steps. First, a CNE-null AcMNPV was obtained from bMON14272 by the substitution of the CNE with *CAT* using the λ Red recombination system; the resulting intermediate bacmid was named vAc^CNE-KO^. The CNE-repair bacmid was constructed according to the Bac-to-Bac protocol by the transformation of the transfer vector carrying the CNE-*egfp* block into DH10B cells that already contained the bacmid vAc^CNE-KO^. Cassette transposition from the transfer vector into the bacmid genome resulted in CNE*-egfp* block incorporation into the *ph* locus located approximately 8 kb away from the original CNE position.

For accuracy, it is important to note that the sequence replaced by *CAT* in the vAc^CNE-KO-EGFP^ and vAc^CNE-KO-REP-EGFP^ genomes was not the 156-bp CNE itself but the CNE flanked by an upstream 5-bp sequence and a downstream 10-bp sequence (Figure S4). Accordingly, the replacement of the CNE with *CAT* resulted in the elimination of 165 bp of the 5′ region of *Ac152* together with 6 adjacent bp and, correspondingly, the 171-bp conserved portions of each of three transcribed sequences. The sequence inserted into the *ph* locus of vAc^CNE-KO-REP-EGFP^ was represented by the CNE flanked by a 19-bp sequence upstream and a 25-bp sequence downstream. This 200-bp insert appears to be the region shared by the putative protein-coding gene *Ac152* and the two overlapping ncRNA genes transcribed in opposite directions.

As the baculovirus fusion protein gp64 is indispensable for the virus life cycle [69], the gp64 gene was eliminated from the vAc^EGFP^ genome to obtain a non-infectious bacmid for investigating the complementation of the infectivity-deficient mutations. vAc^GP64-KO-GFP^ was constructed by the replacement of *gp64* with *CAT*, analogously to the procedure of vAc^CNE-KO-EGFP^ generation.

### The CNE plays an essential role in AcMNPV pathogenesis, a role not associated with CNE activity as a cis-regulator of adjacent genes

**Figure 4:**
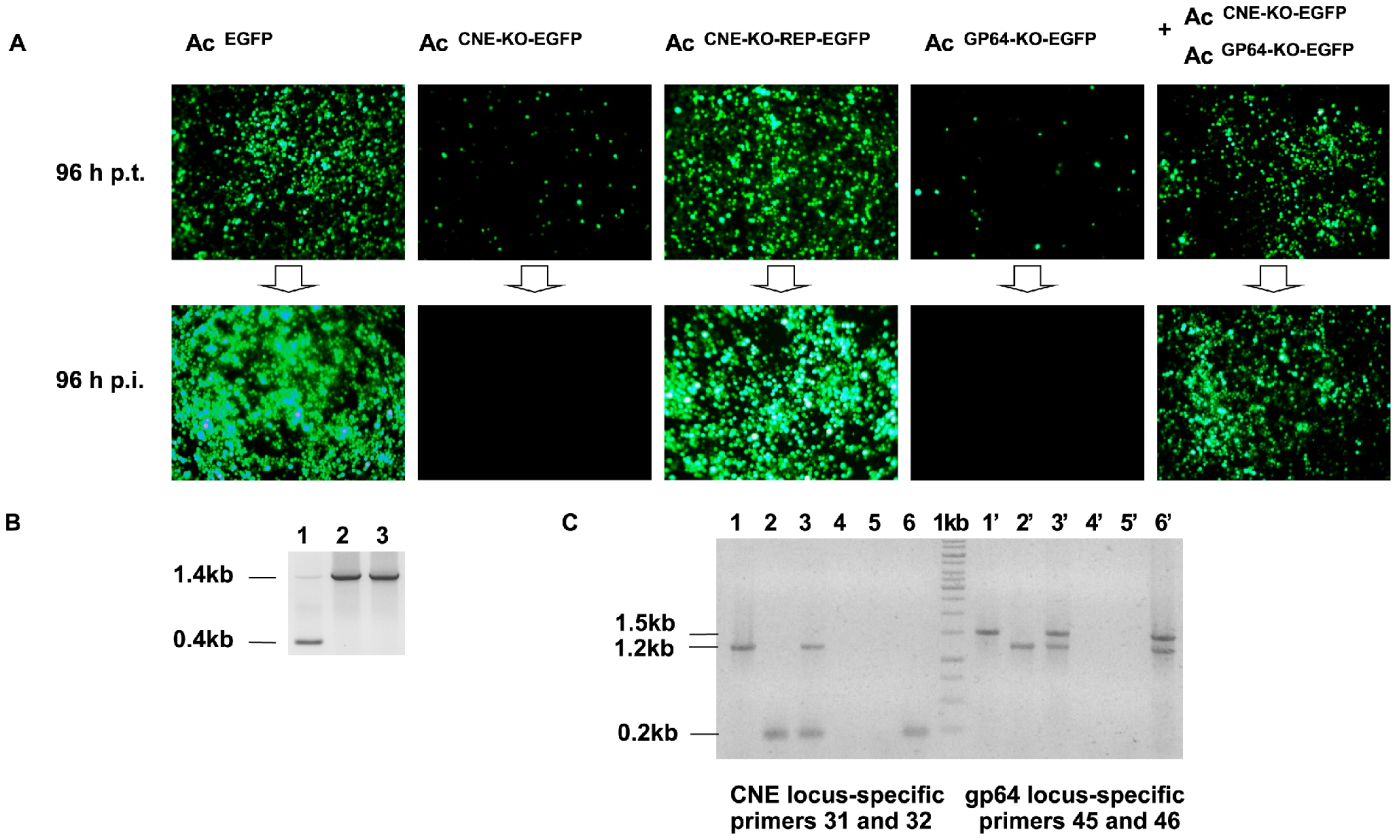
The analysis of the infectivity of vAc^EGFP^, vAc^CNE-KO-EGFP^, vAc^CNE-KO-REP-EGFP^, and vAc^GP64-KO-EGFP^. **A**. Cells transfected with DNA from the indicated constructs (the top row of images) and cells inoculated with the supernatants harvested from the transfected cells (the bottom row of images). The images were captured at 96 h p.t. and at 96 h p.i. **B**. Confirmation of vAc^CNE-KO-REP-EGFP^ infectivity. A 1.4-kbp fragment indicative of the CNE located in the *ph* locus and a 0.4-kbp fragment indicative of the CNE located in its original position were amplified using a mixture of vAc^CNE-KO-REP-EGFP^ and vAc^CNE-KO-EGFP^ DNA as the template in combination with primers 7 and 8 (see Materials and Methods) (line 1). PCR using the same primers and template DNA purified from vAc^CNE-KO-REP-EGFP^-transfected (line 2) or -infected (line 3) cells resulted in the amplification of a 1.4-kbp fragment indicative of the CNE in the *ph* locus. **C**. Analysis of the CNE and *gp64* loci of viral DNA by PCR. The DNA fragments represented on an electropherogram were amplified using the indicated pairs of primers (see Materials and Methods) and template DNA purified from Sf9 cells transfected with vAc^GP64-KO-EGFP^- (lines 1,1′), vAc^CNE-KO-EGFP^ (lines 2, 2′), and vAc^GP64-KO-EGFP^+vAc^CNE-KO-EGFP^ (lines 3, 3′) and from Sf9 cells inoculated with supernatants harvested from the above-mentioned cells lines 4 and 4′, lines 5 and 5′, and lines 6 and 6′, respectively. The sizes of the fragments marked on the left indicate the following: a 0.2-kbp DNA fragment, identifying an intact CNE locus characteristic of vAc^GP64-KO-EGFP^ and restored vAc^EGFP^; 1.5-kbp DNA fragments, identifying an intact *gp64* locus characteristic of vAc^CNE-KO-EGFP^ and the restored vAc^EGFP^; 1.2-kbp DNA fragments, identifying both CNE and *gp64* loci with native sequences substituted with *CAT* (the 1.2-kbp fragment amplified by the CNE-specific primers is indicative of vAc^CNE-KO-EGFP^, and the 1.2-kbp fragment amplified by the *gp64* locus-specific primers is indicative of vAc^GP64-KO-EGFP^).

A transfection/infection assay was performed to assess whether the virus infection cycle was affected by the CNE deletion and whether CNE activity was dependent on the CNE position within the AcMNPV genome. Semi-confluent monolayers of Sf9 cells were transfected with each of three bacmids, vAc^EGFP^, vAc^CNE-KO-EGFP^, or Ac^CNE-KO-REP-EGFP^, and the transfected cells were examined using fluorescence microscopy at different times post-transfection (p.t.) to follow the development of the infection process. Similar transfection efficiencies were achieved, as approximately 20% of the fluorescent cells were detected in each monolayer at 48 h p.t., irrespective of the transfected bacmid. Further observation revealed that the number of fluorescent cells in the vAc^CNE-KO-EGFP^-transfected monolayers remained unaltered at 96 h p.t., whereas nearly 100% of the cells emitted fluorescence in the vAc^EGFP^- and vAc^CNE-KO-REP-EGFP^-transfected monolayers at this time point (Figure 4A). The cells were then incubated for up to 3 days, and fluorescence monitoring was performed daily. No increase in the amount of cells emitting green light in the vAc^CNE-KO-EGFP^-transfected monolayers was observed during this time, clearly reflecting the inability of the CNE-deficient DNA to generate infectious virus particles capable of spreading the infection from the initially transfected cells. In contrast, the increase in the number of fluorescent cells in the vAc^CNE-KO-REP-EGFP^-transfected monolayers up to 96 h p.t. undoubtedly suggested virus transmission from cell to cell. To confirm the obtained results, fresh Sf9 cell monolayers were inoculated with supernatants harvested from the transfected cells. The subsequent fluorescence-based monitoring of the infection process revealed an absence of egfp-expressing cells in the monolayers incubated with the vAc^CNE-KO-EGFP^ supernatants at any time point post-inoculation (p.i.) and an increase in the number of egfp-positive cells up to 96 h p.i. in the monolayers incubated with the vAc^EGFP^ and vAc^CNE-KO-REP-EGFP^ supernatants, results that are consistent with the data from the transfection assay.

The transfection/infection assay data clearly demonstrated the inability of vAc^CNE-KO-EGFP^ to spread the infection. Regardless, these data were not considered as sufficient to postulate the infectious nature of vAc^CNE-KO-REP-EGFP^ due to the following reason. Because the CNE-containing fragment replaced by *CAT* in the vAc^CNE-KO-REP-EGFP^ genome is 29 bp shorter than the CNE-containing fragment inserted into the *ph* locus, identical 14- and 15-bp sequences flank both the transposed *CAT* and the CNE located within the heterologous locus. Due to the recombinogenic nature of baculovirus DNA [70] [71], identity between the sequences flanking both inserts could be considered a prerequisite for the generation of recombinant genomes in which the CNE would be reinserted in its original position by homologous recombination. Although 14- and 15-bp flanking sequences appear to be too short to provide efficient genome fragment exchange, the low-frequency occurrence of recombinant genomes during viral replication in cell culture cannot be absolutely excluded. Thus, to assess whether the infectious virus harvested from vAc^CNE-KO-REP-EGFP^-transfected cells was not a recombinant virus carrying the CNE in its original position, PCR-based analyses of the viral DNA were performed. Primers 7 and 8 (see Materials and Methods), located upstream and downstream of the *CAT* insert, respectively (Figure 3), were used in the reaction. Only a 1.4-kb fragment, indicative of the originally constructed vAc^CNE-KO-REP-EGFP^ genome, was amplified from the viral DNA template extracted from both the vAc^CNE-KO-REP-EGFP^-transfected cells and the cells inoculated with the corresponding supernatant (Figure 4B). Multiple repetitions of the PCR-based test failed to detect a 0.4-kb fragment, which indicates that the CNE had reverted to its original position. These data confirmed that the observed recovery of infectivity of the CNE-deficient DNA was caused by the CNE insertion into the heterologous genome locus.

Thus, the data presented provide evidence of the non-infectious nature of vAc^CNE-KO-EGFP^ and the infectious nature of vAc^CNE-KO-REP-EGFP^. The fact that the elimination of the CNE from the *ie2* promoter-proximal region does not abolish vAc^CNE-KO-REP-EGFP^ infectivity suggests that its role as an upstream activating region of *ie2*, established by previous research [58], is non-essential for virus propagation in cell culture. Accordingly, it can be concluded that an as-yet-undefined essential function other than transcriptional cis-regulation must be attributed to the CNE. Because a CNE-containing fragment was used for the generation of a repaired virus rather than the entire CNE, the data do not exclude a possible engagement of the 19-bp 5′-adjacent and 25-bp 3′-adjacent sequences in CNE activity.

### The CNE includes an essential *nfe* directly involved in DNA transaction(s)

As noted previously, *Ac152*, which overlaps the AcMNPV CNE, should be transcribed and, probably, translated; therefore, it appears likely that the lack of infectivity of CNE-knockout DNA may be due to *Ac152-*associated product (RNA or protein) deficiency. However, the infectious nature of vAc^CNE-KO-REP-EGFP^ is consistent with the conclusion that even if *Ac152* represents a coding sequence, its product is not essential for viral pathogenesis (vAc^CNE-KO-REP-EGFP^ is unlikely to express the active *Ac152* product because it carries a truncated gene consisting of 179 bp of *Ac152* preceded by only 21 bp of its 5′-proximal region that is not expected to enclose a minimal promoter). Similarly, if TSS^131594^-directed transcription occurs, it should be non-essential for viral infectivity, as TSS^131594^-deficient vAc^CNE-KO-REP-EGFP^ remains viable.

It is remarkable, however, that the inserted CNE includes the TSS^132300^ and the length of the 5′-region immediately adjacent to the TSS^132300^ (108 bp) is large enough to include a minimal promoter. Hence, it is possible that the 92-bp conserved part of the TSS^132300^-related ncRNA could be expressed and still maintain its biological activity in vAc^CNE-KO-REP-EGFP^. From this, the question arises whether the CNE includes an essential *nfe* (a sequence engaged directly in an essential DNA transaction) or whether the essential activity of the CNE-containing locus is mediated by the CNE-related expression product, ncRNA. A complementation assay was applied to clarify this issue. The objective of the experiment was to explore whether a CNE defect could be rescued by trans-complementation with a helper virus harboring an intact CNE.

Two non-infectious bacmids, CNE-deficient vAc^CNE-KO-EGFP^ and *gp64*-deficient vAc^GP64-KO-EGFP^, were used in the experiment, and both the ability of vAc^CNE-KO-EGFP^ to complement vAc^GP64-KO-EGFP^ and its ability to be complemented by vAc^GP64-KO-EGFP^ were examined. During the experiment described in the previous section, the bacmids vAc^CNE-KO-EGFP^, vAc^GP64-KO-EGFP^, and their mixture, were used to transfect Sf9 cells. The number of cells emitting fluorescence was found to be comparable between all transfected monolayers at 48 h p.t. A further inspection of the samples at 96 h p.t. revealed an unaltered number of fluorescent cells in the monolayers that were transfected with each of the deletion mutants (vAc^GP64-KO-EGFP^ or vAc^CNE-KO-EGFP^) alone and a greatly increased number of fluorescent cells in the monolayers that were co-transfected with the vAc^GP64-KO-EGFP^ and vAc^CNE-KO-EGFP^ mixture. The subsequent passaging of the inocula harvested from the transfected cell monolayers onto fresh Sf9 cells verified that the inoculum from the mixed transfected cells contained infectious virus (Figure 4A). It was predictable that the observed “rescue of infectivity” should be a result of the recovery of the wild-type phenotype (vAc^EGFP^) caused by recombination between the *gp64*- and CNE-deficient mutant genomes and as a result of the viability of vAc^GP64-KO-EGFP^ complemented with gp64 expressed from the vAc^CNE-KO-EGFP^ genome. To confirm this and to address the question of whether CNE deficiency was also complemented in trans, viral DNA was purified from both the transfected and infected cells and subsequently tested for the presence of different genotypes. Two pairs of primers (31, 32 and 45, 46) were designed (see Materials and Methods) to amplify fragments that could be indicative of CNE-deficient, *gp64-*deficient, CNE-intact, and *gp64-*intact genomes (Figure 3). The PCR analysis demonstrated that only CNE-intact genomes were present in the cells inoculated with the viruses that originated from the mixed transfection; only the 0.2-kbp fragment indicative of the intact CNE was amplified, and there was no evidence of the 1.2-kbp fragment, which is indicative of a CNE-deficient genome, on the electropherogram (Figure 4C, line 6) in three repeated experiments. As expected, the 1.2-bp fragment indicative of vAc^GP64-KO-EGFP^ was amplified (Figure 4C, line 6′) together with the 1.5-kb fragment indicative of a *gp64*-intact virus. Taking into consideration the amplification pattern discussed above (Figure 4C, line 6), the 1.5-kbp fragment could not be attributed to the presence of the vAc^CNE-KO-EGFP^ genomes in the DNA sample analyzed. Plaque assays confirmed the presence of vAc^EGFP^ in the virus population that originated from the mixed transfection (data not shown), thus explaining the amplification of the 1.5-kbp fragment.

Therefore, data from the complementation test provided evidence that the rescue of viral infectivity observed under the conditions of mixed transfection occurred due to the complementation of the deletion mutation vAc^GP64-KO-EGFP^ by exogenous gp64 and to the recombination-dependent recovery of vAc^EGFP^ and that the defect in the production of infectious vAc^CNE-KO-EGFP^ could not be trans-complemented. These results convincingly demonstrate that the AcMNPV CNE essential activity is not mediated by any trans-factors encoded by it or by the CNE trans-activity itself. Thus, the AcMNPV CNE can be defined as a sequence that includes an essential *nfe*, a genomic element whose non-coding content is directly involved in certain essential DNA transaction. The recovery of infectivity of the CNE-deficient virus by inserting the CNE into the heterologous genomic locus demonstrated that this *nfe* is a genomic element that plays an essential role in the AcMNPV life cycle independently of its genomic location. It should be emphasized that although the genomic segment containing the CNE together with its 19-bp 5′-proximal and 25-bp 3′-proximal regions was sufficient for an essential *nfe*, the possible involvement of additional sequences adjacent to the CNE in modulating its essential activity cannot be excluded.

## Discussion

This study postulates the existence of the CNE, a conserved, uniquely structured element of the *Alphabaculovirus* genome. The CNE can be defined as a nearly symmetrical sequence of 154–157 bp in length that includes a single central DS and is terminated at both ends by DSs, which can be regarded as imperfect copies of each other. Highly conserved positions of both DSs and other CNE characteristic components, such as TAT-containing nucleotide clusters, also characterize the newly discovered element. The data from earlier studies of sequences spanning the AcMNPV CNE suggest that the CNE represents the conserved region of overlapping transcription, it operates as a transcriptional activator, and its DSs function as protein-binding motifs [56] [58] [60]. This study demonstrates that the AcMNPV CNE also includes some additional *nfe* absolutely required in the viral life cycle. Taken together, all these findings provide a more comprehensive view of the conservation, structure, and significant role of the newly identified element.

### Polyfunctionality of the CNE

The AcMNPV CNE is located between two divergently transcribed immediately early genes, *ie2* and *pe38*. It is generally accepted that the expression activities of each of two closely spaced divergently transcribed genes may be interdependent, such that transcription from one promoter influences the transcription from other promoter [72] [73]. The detection of several genes (ncRNA genes and putative protein-coding gene) spanning the *ie2-pe38* intergenic region [60] (Figure S4) strongly suggests that RNA interference [74] can also be involved in the regulation of both *ie2* and *pe38* expression. It should also be noted, that the *ie2-pe38* intergenic region contains numerous binding sites for some proteins, the true and putative regulators of *ie2* and *pe38* promoter activities [56] [61] (Figure S4). Taken together these facts these facts suggest the existence of a very complex regulatory mechanism driving the coordinated expression of *ie2* and *pe38*. The AcMNPV CNE as a part of *ie2-pe38* intergenic region includes three of the six protein-binding sites found in this region, and represents the conserved part of the overlapping genes (Figure S4). Transient expression assays aimed to investigate the effect of deletions in the 5′ untranslated region of *ie2* demonstrated that the sequence recognized as the CNE in this study appeared to be an upstream activator of the core *ie2* promoter. However, as the CNE may be expected to play in concert with other regulatory elements of the *ie2-pe38* intergenic region, its role in *ie2-pe38* expression may be more complicated.

The detection of CNEs in all *Alphabaculovirus* genomes indicated a high probability that this new sequence plays an indispensable role in the *Alphabaculovirus* infection cycle. Transfection-infection assays showed an inability of the CNE-knockout AcMNPV-based bacmid to spread the infection, thereby providing experimental evidence of an essential role of the CNE in virus propagation. Regardless of whether the CNE regulates the activity of one or both immediately early promoters, this cis-regulatory activity would be expected to correlate with its essential role. However, further assays refuted the suggestion regarding the association of the essential role of the AcMNPV CNE with its cis-regulatory activity, as the CNE repair bacmid harboring CNE inserted into a heterologous region was shown to be infectious. Additionally, the complementation test demonstrated that an essential function of the AcMNPV CNE cannot be determined by the activity of any trans factors (ncRNAs or proteins) encoded by sequences that overlap the CNE. The conclusion regarding the existence of an additional DNA transaction performed by the CNE, which could not be assigned to the cis-regulation of the immediately early genes, was based on the aforementioned experiments, and this conclusion was consistent with the data available from two earlier studies. First, the evidence that the CNE is implicated in two temporally distinct processes arises from the observation of Krappa and co-workers [56] that, although the AcMNPV DSr and DSl bind proteins of cellular origin at an early stage of infection, the DSc-protein complex may be detected at 40 h p.i. (the protein partners of DSc are expected to be of viral origin because the cessation of host protein synthesis occurs at this time [75]). In other words, the polyfunctional nature of the CNE can be considered to be a derivative of different specializations of the DSs composing it. Second, as the linearization of the ManeNPV genome mediated by the CNE sequence is unlikely to be attributed to the CNE transcriptional regulatory activity, it is logical to suggest that a DNA double-stranded “break” is associated with CNE involvement in some other DNA process related to the conversion of the ManeNPV genome form. However, it remains unclear whether this suggestion concerns only the ManeNPV CNE or whether it is applicable to the CNEs of other alphabaculoviruses. The analysis of some uncharacterized phenomena observed earlier by Oppenheimer and Volkman [76] suggests an answer to this question. The resolution of single-cut, nuclease-digested replicative intermediates of AcMNPV DNA by pulsed field electrophoresis demonstrated the existence of a short 14-kbp fragment in the pool of full-size genomes, which may be indicative of genomes with defined free ends. Oppenheimer and Volkman reported that the mapping of a 14-kbp fragment terminus resulted in the identification of the genome locus corresponding to the *ie2 - pe38* intergenic spacer, i.e., the linearization site of the AcMNPV genome was localized to the aforementioned region. The comparison of the location of the AcMNPV *ie2-pe38* intergenic region (132083–132526) to that of the AcMNPV CNE (132228–132383) led to the finding that the AcMNPV CNE is enclosed within this region. Although the position of the linearization site in the AcMNPV *ie2-pe38* intergenic region was not precisely determined by the authors of the work, the location of the linearization site within the ManeNPV CNE strongly suggests that the analogous site may be a requisite of the AcMNPV CNE. Thus, the conversion of the genome form associated with the CNE may be a feature shared by alphabaculoviruses. It can be assumed that the specificity of the ManeNPV-Anpe cell system is most likely not attributable to the generation of DNA linear forms but rather to the abnormal accumulation of these forms to easily detectable levels during ManeNPV DNA propagation in Anpe cells. The implication of the ManeNPV CNE in genome form conversion allows the hypothesis that this element may be involved in process associated with DNA replication and/or recombination and/or cleavage because these transactions should inevitably result in short-lived linear intermediate genomes. The localization of the linearization site in the AcMNPV genome is required to determine whether this hypothesis can be extended to the AcMNPV CNE.

### Originality of the CNE

It is generally accepted that, in contrast to the coding genomic elements, *nfes* do not commonly exhibit an evolutionary tendency toward extended sequence conservation: the general *nfe* structure, rather than its overall sequence, appears to be subjected to negative selection pressure. Accordingly, there is a high probability that the regions of extended homology between the non-coding sequences of representatives of same taxonomic group are merely a result of background conservation caused by the short evolutionary distances between the compared species. However, this is not the case with the *Alphabaculovirus* CNE, as may be deduced from the following. As protein-coding sequences are under strong evolutionary constraint, the average level of amino acid identity between proteins shared by two genomes appears to be a robust measure of the whole-genome level of relatedness between the species being compared. This level of identity was found to be relatively low for particular pairs of *Alphabaculovirus* species, exemplified by HearSNPVG4-BmNPV and LyxyMNPV-AcMNPV, which exhibit 41% and 33% amino acid identity, respectively [77] [44]. In addition, a high variability in GC content and gene number (ranging from 118 to 169 genes) across *Alphabaculovirus* genomes, a relatively low proportion of homologous genes shared by all alphabaculoviruses (among them are 37 core and 14 *Alphabaculovirus*-specific genes) compared to the entire gene content of each genome [9], and a large number of rearrangements in the genomes of particular alphabaculoviruses [78] suggest that the *Alphabaculovirus* genus includes highly diverged species. Therefore, background conservation is not expected to be significant between the CNEs of distantly related alphabaculoviruses. The detection of highly variable regions along the CNE consensus sequence (Figure 2) is in accordance with the above conclusion.

The recognition of a CNE in the genomes of distantly related species on the basis of the primary structure makes the CNE different from other baculovirus *nfes*, promoters, non-*hr oris*, and *hrs*. A/G/TTAAG and CAGT motifs are highly conserved transcriptional initiators of late and early baculoviral promoters respectively [79] [80], however the overall nucleotide sequences of baculoviral promoters are variable even between orthologous genes of quite closely related viruses. Non-*hr oris* share common elements (repeated sequences, AT-rich regions, and recognition motifs for transcription factors) but do not exhibit a tendency of sequence conservation between *Alphabaculovirus* species [16] [17] [18]. All baculoviral *hrs* consist of similar building blocks represented by stretches of direct repeats centered by palindromic sequence [7] [21] [10], with only short core palindromes exhibiting homology when the *hrs* of distantly related alphabaculoviruses are compared [34]. Similar to baculoviral *hrs*, many of the diverse viral non-coding functional elements exemplified by the herpesvirus packaging signal [81], coronavirus packaging signal [82], and poxvirus signal of DNA concatamer resolution [83] are represented by evolutionary conserved short single sequence motifs (regions of structural significance or binding sites for enzymes) embedded in sequences, with their lengths and nucleotide contents varying widely among the members of a virus family. In the genomes of some viruses, diverse *nfes* are concentrated within specifically organized regions; consequently, short conserved motifs that specify different elements may be found in close proximity to each other in those regions. The most prominent examples of such polyfunctional regions include the following: retroviral LTRs (long terminal repeats), which appear to be highly conserved with regard to the composition and arrangement of short conserved motifs (i.e., identifiers of the integration signal, signal of transcription regulation, and signal of RNA processing) but are not conserved with regard to the length and overall nucleotide sequence [84]; and the *Alphapapillomavirus* LCRs (long control regions) of variable length, which share numerous short sequence motifs (identifiers of *ori* and cis-regulatory elements) but do not exhibit long stretches of sequence similarity when different alphapapillomavirus types are aligned [85]. The distinct protein recognition motifs grouped together within the limited genomic region may also specify a single functional unit in the genomes of certain viruses, e.g., the attP sites of some phages (sequences that are engaged in the process of phage genome integration into the host chromosome) consisting of recombinase recognition motif(s) supplemented with recognition motif(s) for accessory proteins [86]. Accordingly, the CNE can be regarded as a sequence that consists of several conserved motifs arranged in a certain order relative to each other that does not appear to be extraordinary compared to known viral *nfes* and known polyfunctional genomic regions consisting of several *nfes*. However, to my knowledge, there is neither a known viral *nfe* nor a known viral genomic region composed of several *nfes* that exhibits an evolutionary tendency toward preservation of both overall sequence length and motif composition and arrangement. Thus, the location of several closely spaced, conserved motifs at strictly conserved positions along the CNE length, which determines the strictly fixed length of the CNE and its easy recognition on the basis of the overall sequence homology, makes the CNE unique among the known viral *nfes*.

Whether the absolute linkage between the several *nfes* is of any biological significance remains to be determined. The fact that the AcMNPV CNE is enclosed in the genomic locus shared by the overlapping ncRNA genes cannot be ignored in the context of the speculations regarding this item. This locus is likely to encode the biologically relevant RNA regions whose genetic variations are constrained by evolution through natural selection. It is possible that the conservation of such relevant RNA-coding sequences simply determines the conserved arrangement of the conserved *nfe* motifs, the parts of these sequences (and/or vice versa).

### CNE structure

It is well established that the target motifs of many DNA-binding proteins exhibit nucleotide variability and the conserved nucleotides of such slightly mismatched motifs were shown to constitute the loci of direct DNA-protein interactions [87]. Taking into account the established roles of AcMNPV DSs as protein-binding sites, the core arms of DSs (hexameric IRs, requisite of both DSl and DSr, and nanomeric IRs, requisite of DSc) appear to be good candidates for the degenerate motifs directly involved in the binding of two identical cooperatively acting proteins or, alternatively, in the binding of homodimeric protein subunits. The resemblance between the left arm of the DSc (GtAGACTaT) and the conserved nucleotide cluster 2 (gTAgTaTAT), as demonstrated by the CNE consensus (Figure 2A), suggests that the latter may represent a third degenerate motif for the binding of the DSc-associated protein. It also appears reasonable to suggest that the TAT conservation at two additional positions along the CNE length may also reflect this triplet embedded in the motif for the binding of the above-mentioned protein. A comparative analysis of the TAT-containing 9-bp sequences found at all 5 positions (including the complement of the right DSc arm) using the WebLogo tool failed to identify significantly conserved nucleotides, except for TAT (data not shown). This result, however, may be considered to be in agreement with the hypothesis that TAT-containing sequences may represent a variable motif similar to the highly degenerate protein-binding motifs frequently found in the genomes of different origins [88] [89] and in viral genomes in particular. Some examples include some bacteriophages attPs, which enclose the target site for a site-specific recombinase, clustered with the additional multiple degenerate binding sites for recombinase and/or for accessory proteins [86] [90] [91] and the Epstein-Barr virus *ori P*, which contains 26 18-bp motifs that are slightly variable with regard to their nucleotide composition and affinity for the EBNA1 initiator protein [92].

An alternative, but not mutually exclusive hypothesis, is that the conserved nucleotide clusters of the CNE might represent structurally significant elements under negative selection. It is now widely recognized that DSs (palindromes) appear to be the major participants in many biological processes, and their functions are often associated with their capacity for intra-strand base-pairing, which results in the formation of alternative DNA structures called hairpins (if one DNA strand is engaged in the process) and cruciforms (if both strands participate in the process) [93]. Hairpins also represent the dominant secondary structure element in many RNAs, where they define nucleation sites for folding, determine tertiary interactions in RNA enzymes, protect mRNAs from degradation, and are recognized by RNA-binding proteins [94]. The CNE DSs exhibit an evolutionary tendency toward the preservation of a palindromic structure despite the turnover of the nucleotides framing the core DSs (Figure S5). This tendency suggests that the CNE is a type of sequence that is potentially capable of structural transitions, and, consequently, the conservation of both DSs and TAT-containing sequences may reflect the role of these sequences in cruciform/hairpin extrusion and/or maintenance in DNA and/or RNA. The AT-rich sequences were shown to facilitate DNA structural transitions as they have the common feature of being easily unwound [95] [96] [97]. Accordingly, the correlation between the CNE extrusion capacity and the high AT content of the CNE may be expected.

## Conclusion

The newly identified, uniquely structured baculovirus genomic element, CNE, appears to be an *Alphabaculovirus* genus-specific marker. Based on the results of the present study and data available in the relevant literature, the AcMNPV CNE can be defined as a module consisting of several functionally autonomous but structurally dependent units rather than a single functional unit. At least two *nfes*, a cis-regulator of early transcription that is non-essential for virus pathogenesis and an essential *nfe* with undefined function, are enclosed in the CNE, whereas the CNE itself constitutes the conserved part of the region of overlapping transcription. Work is currently underway to elucidate the function of the essential *nfe* and its underlying mechanism.

## Materials and Methods

### Bioinformatic analysis

The search for homology between the ManeNPV genome fragment and the sequences available in databases was performed using the BLASTN algorithm of the BLAST homology search software. The ClustalW alignment tool [98] was used to estimate the identity levels between the 38 homologous sequences revealed and to extract a consensus sequence, and the WebLogo tool [57] was used to represent the similarities and differences within the CNE sequences in a visually appealing form.

### Viruses and cells

The AcMNPV recombinant bacmids were derived from the commercially available bacmid bMON14272 and propagated in *Escherichia coli* strain DH10B (Invitrogen Life Technologies). The bacmid-based recombinant viruses were propagated in Sf9 cells maintained in serum-supplemented TC100 medium at 27°C, as previously described [99].

### Construction of recombinant bacmids

The commercially available Bac-to-Bac system (Invitrogen Life Technologies) was used to introduce the *egfp* gene into bMON14272. Briefly, *egfp* was excised from pC1-EGFP by digestion with *Nhe*I and *Kpn*I and ligated to the *Nhe*I-*Kpn*I-digested transfer vector pFastBacDual under the control of the late *p10* promoter. The recombinant bacmid vAc^EGFP^ was generated by Tn7-mediated transposition of the *p10* promoter-*egfp* block into the parental bacmid backbone, as previously described [68].

Two CNE-knockout bacmids, bMON14272-based and vAc^EGFP^-based bacmids, were generated using the λ Red-mediated recombination approach routinely used for the selective knockout of baculoviral genes [100] [101] [102]. First, DH10B cells containing bMON14272 and DH10B cells containing vAc^EGFP^ were transfected with pKD46, a plasmid carrying the genes involved in the production of recombination-stimulating factors. A *CAT* cassette with the CNE-flanking regions was amplified using the primers 27-(5′-ACGACTGATAAGACAATA GTGGTfGGGGGAACTTGCCAGGCAATTTCACCAGCGTTTCTGG-3′) and 28-(5′-GGGGG ACAGATAACAGAAACTGCAGCCTGTGATATGATAAATAAGGGCACCAATAACTGC-3′) with pBR325 as the template. These primers contained 42 and 41 bp (underlined) homologous to the CNE-flanking regions. The amplified fragment was electroporated into DH10B cells harboring bMON14272 or vAc^EGFP^ together with pKD46. The electroporated cells were incubated at 37°C for 4 h in 1 ml of SOC medium and plated on agar medium containing 8 µg of chloramphenicol and 50 µg of kanamycin per ml. The plates were incubated at 37°C overnight, and the colonies resistant to the antibiotics were selected. The primers 7-(5′-CGGAATTCGGCTGGGCTGGTAGGAT-3′) and 8-(5′-CGGATCCTTTGCTTATTGGCAGGC-3′) were used for a PCR-based test to confirm the insertion of the *CAT* gene into the CNE locus. CNE-deficient recombinant bacmids (one of which contained *egfp*, whereas the other did not) were named vAc^CNE-KO-EGFP^ and vAc^CNE-KO^, respectively.

The gp64-knockout bacmid, named vAc^GP64-KO-EGFP^, was constructed analogously to vAc^CNE-KO-EGFP^. The *CAT-*containing fragment was generated by using the primers 33-(5′-TTA CAAAGTTAACTACATGACCAAACATGAACGAAGTCAATTTCACCAGCGTTTCTGG-3′) and 34-(5′-TAAATCAGTCACACCAAGGCTTCAATAAGGAACACACAATAAGGGCACCA ATAACTGC-3′). The primers 45-(5′-CATGACCAAACATGAACGAAGTC-3′) and 46-(5′-C AGTCACACCAAGGCTTCAATAAG -3′) were used for a PCR-based test to confirm the *CAT* gene placement into the *gp64* locus.

The CNE-repaired bacmid vAc^CNE-KO-REP-EGFP^ was constructed by insertion of an AcMNPV genome fragment comprising the CNE flanked by 19 and 25 bp at its 5′ and 3′ ends, respectively, into the vAc^CNE-KO-EGFP^ *ph* locus. The Bac-to-Bac system mentioned above was used for this purpose. The CNE-containing fragment was amplified using the primers 31-(5′-AAC CCGGGACTTGCCAGGCA-3′) and 32-(5′-GACCCGGGCTGTGATATGAT-3′). The amplified fragment was digested with *Sma*I and inserted into the pFastBacDual-EGFP Bst1107 cloning site located in the *ph* locus. The resulting recombinant transfer vector was used for the Tn7-mediated transposition of the *egfp*-CNE block into vAc^CNS-KO^. PCR amplification with insert-specific primer 31 and bacmid-specific M13 reverse primer (Invitrogen Life Technologies) was performed to confirm the insertion of the CNE into the *ph* locus.

### Transfection/infection assay

A transfection/infection assay [101] was performed to examine the ability of the recombinant bacmids to infect Sf9 cells. Bacmid DNA was extracted from *E. coli* cells in strict accordance with the Bac-to-Bac manufacturer's instructions (Invitrogen Life Technology). The transfection of bacmid DNA into insect cells was performed using the DOTAP transfection reagent (Roche Applied Science). Briefly, 5 µl of bacmid DNA solution and 8 µl of DOTAP were mixed with 35 µl and 72 µl of HBS buffer, respectively, and gently shaken. The two solutions were then combined, mixed by pipetting, and incubated for 30 min at room temperature. The DNA-DOTAP complex was diluted with 360 µl of TC100 serum-free medium and layered onto a monolayer of Sf9 cells at approximately 50-60% confluence in 35-mm Petri dishes. After 5 h of incubation, the DNA-DOTAP-containing supernatant was replaced with fresh medium, and the incubation was continued. The transfected monolayers were inspected with a fluorescence microscope at 48 and 96 h p.t. to determine the percentage of cells emitting fluorescence. At 96 h p.t., the cells were harvested for DNA extraction, and the supernatants were harvested and used for the inoculation of fresh monolayers. The inoculated monolayers were examined with a fluorescence microscope at 96 h p.i., and the cells were harvested for DNA extraction.

### DNA purification, treatment, and identification

Viral DNA was purified from the transfected/infected cells as previously described [103]. The DNA purified from the cell monolayer grown on 35-mm Petri dishes was dissolved in 60 µl of TE buffer. A 20-µl aliquot of DNA was treated with *Dpn*I in a volume of 50 µl to remove possible contaminating DNA that was used for the transfection. (Methylation-dependent *DpnI* cleaves the bacmid DNA originating from bacterial cells and leaves intact the bacmid DNA replicated in insect cells.) The *Dpn*I-treated DNA was ethanol-precipitated, dissolved in water, and subjected to a PCR-based analysis. For the analysis of the CNE locus, primers 31 and 32, described in the previous section, were used to amplify a 0.2-kbp fragment, as an identifier of the intact CNE locus, or a 1.4-kbp fragment, as an identifier of the CNE-deficient locus. For the analysis of the gp64 locus, primers 45-(5′-ACATGACCAAACATGAACGAAGTC-3′) and 46-(5′-CAGTCACACCAAGGCTTCAATAAG-3′) were designed to generate a 1.5-kbp fragment, which was indicative of intact gp64, or a 1.4-kbp fragment, which was indicative of a gp64-deficient genotype.

## Supporting Information

Figure S1 Cloning, mapping and sequencing of the BamHI-D fragment of the ManeNPV genome. (a) Physical map of pMB-D, the recombinant plasmid constructed by insertion of the ManeNPV genome BamHI-D fragment into the pBR325 plasmid; (b) BamHI-D fragment map showing the location of the conserved element, the linearization site, the hoar gene and an unidentified ORF; (c) the conserved element sequence.
(PDF)

Figure S2 The HindIII-BglII genome fragment of the *Malacosoma neustria* nucleopolyhedrovirus (ManeMNPV). The letters on a green background identify the *hoar* sequence, on a turquoise background – the ManeNPV-specific ORFs. The start codons are underlined. The CNE sequence is indicated by the red bold letters. The letters on the blue, red, yellow, grey backgrounds mark HindIII, KpnI, PstI, BglII sites respectively.
(PDF)

Figure S3 Multiple CNE alignment. Consensus sequence is shown at the bottom of the alignment. The uppercase letters denote the completely conserved nucleotides, and the lowercase letters denote nucleotides with 50% or greater conservation. The letter “n” indicates that no completely or highly conserved nucleotides were found at the corresponding position. The points mark the positions in which the majority of the aligned sequences contain a gap.
(PDF)

Figure S4 The nucleotide sequence of the AcMNPV genomic region encompassing the CNE. The letters on a green background identify the *ie-2* sequence, on a gray background – the *Ac152* sequence, on a turquoise background – the *pe-38* sequence. The ATG codons are underlined. The CNE sequence is indicated by the lowercase bold letters. Two symmetrical near-identical sequences encompassing protein-binding sites are indicated by blue letters, the third symmetrical sequence encompassing a protein-binding site – by green letters, ie1 target sites – by red letters, GATA-binding site – by orange letters. Three core DSs are underlined and highlighted in blue (DSl, DSr) and green (DSr). The red quotes are used to mark the beginning and end of the CNE-containing fragment that was deleted from the bacmid to obtain vAc^CNE-KO-EGFP^, blue quotes – to indicate the beginning and end of the CNE-containing fragment that was inserted into vAc^CNE-KO-EGFP^ genome to obtain vAc^CNE-KO-REP-EGFP^. The arrowheads marks the transcription start sites (TSSs), the capital letters on a purple background – the first nucleotide to be transcribed. A rhombus marks the polyadenylation signal (PAS), a capital letter on yellow background – the last nucleotide to be transcribed.
(PDF)

Figure S5 The 200-bp CNE-containing sequences of 38 alphabaculovirus genomes. The letters on a gray background marks the left, central and right dyad symmetry elements (DSl, DSc, DSr respectively). The Dsc, DSl, and DSr are extracted from each CNE to demonstrate the extent of their IRs with the respect to the core IRs (core IRs are underlined).
(PDF)

Table S1 Identity scores between 38 alphabaculovirus CNEs.
(PDF)

Table S2 A comparison of the alphabaculovirus genome AT content and the CNE AT content.
(PDF)
