# Supplementary figures and images for: Identification of a Conserved Non-Protein-Coding Genomic Element that Plays an Essential Role in Alphabaculovirus Pathogenesis
Source: PLoS One. 2014 Apr 16;9(4):e95322. doi: 10.1371/journal.pone.0095322 (PMC3989284; doi:10.1371/journal.pone.0095322)

Figure S1

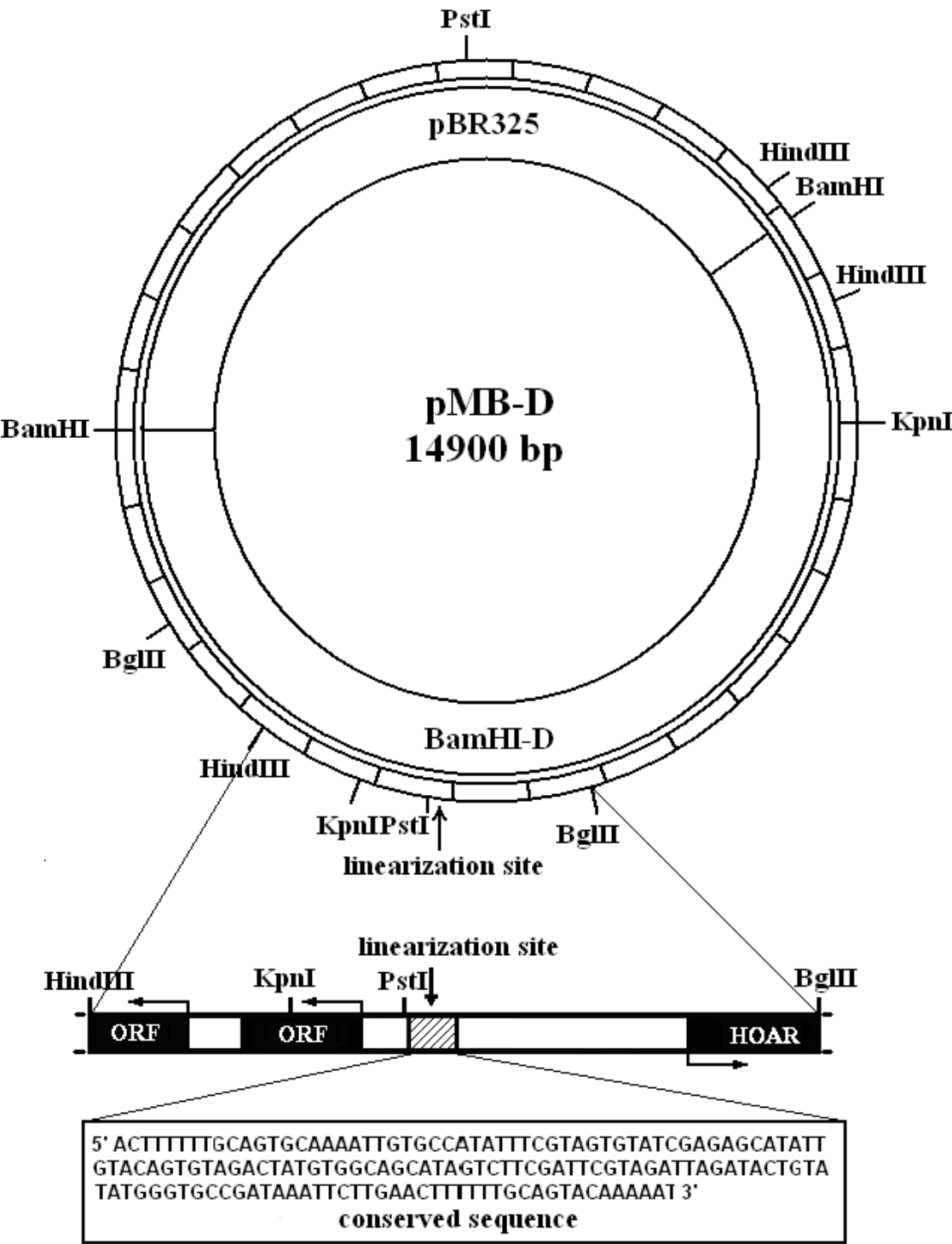

Supplement: Figure S1 — Cloning, mapping and sequencing of the BamHI-D fragment of the ManeNPV genome. (a) Physical map of pMB-D, the recombinant plasmid constructed by insertion of the ManeNPV genome BamHI-D fragment into the pBR325 plasmid; (b) BamHI-D fragment map showing the location of the conserved element, the linearization site, the hoar gene and an unidentified ORF; (c) the conserved element sequence. (PDF) [file pone.0095322.s001.pdf]

FIGURE S4

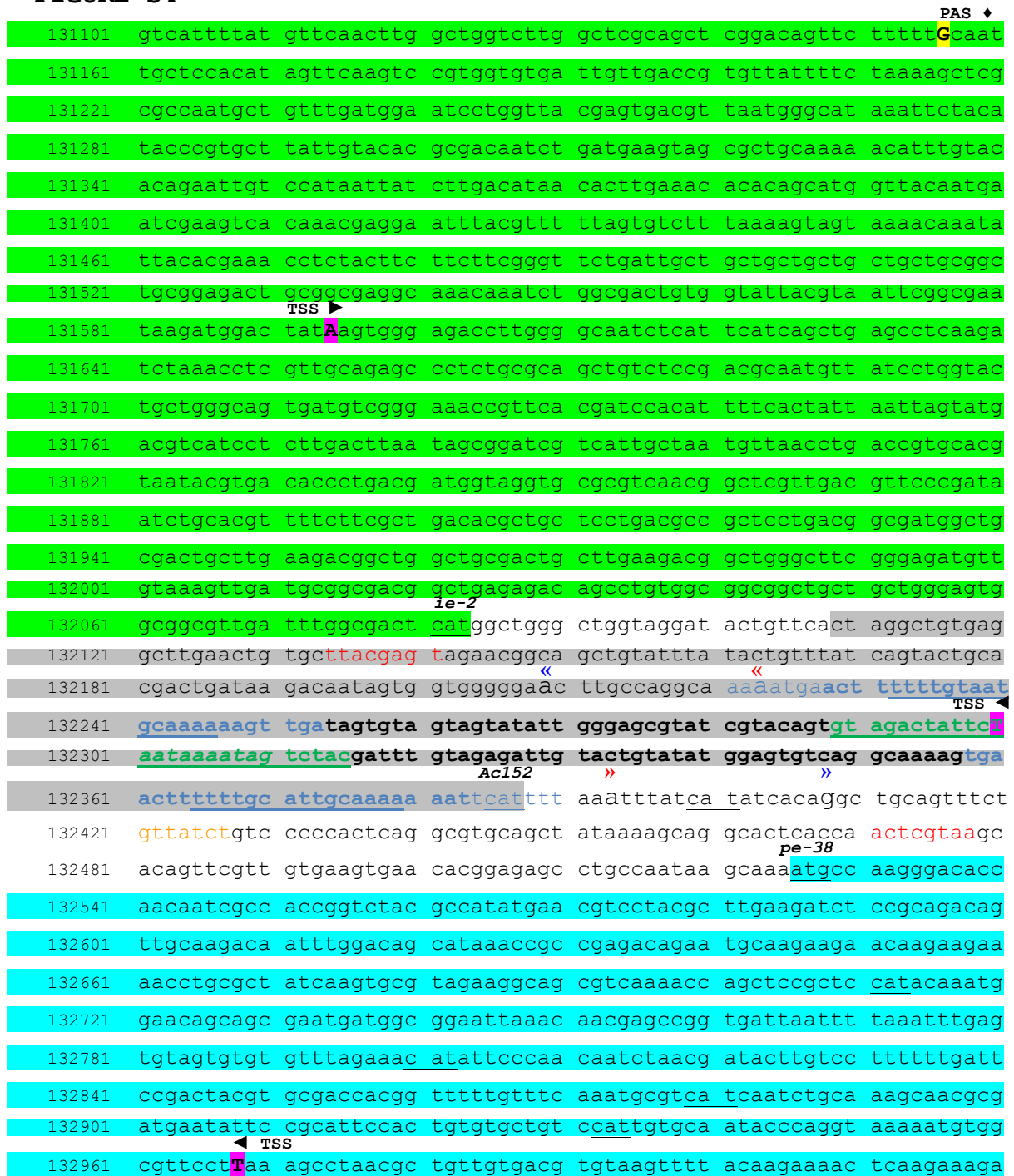

Supplement: Figure S4 — The nucleotide sequence of the AcMNPV genomic region encompassing the CNE. The letters on a green background identify the ie-2 sequence, on a gray background – the Ac152 sequence, on a turquoise background – the pe-38 sequence. The ATG codons are underlined. The CNE sequence is indicated by the lowercase bold letters. Two symmetrical near-identical sequences encompassing protein-binding sites are indicated by blue letters, the third symmetrical sequence encompassing a protein-binding site – by green letters, ie1 target sites – by red letters, GATA-binding site – by orange letters. Three core DSs are underlined and highlighted in blue (DSl, DSr) and green (DSr). The red quotes are used to mark the beginning and end of the CNE-containing fragment that was deleted from the bacmid to obtain vAcCNE-KO-EGFP, blue quotes – to indicate the beginning and end of the CNE-containing fragment that was inserted into vAcCNE-KO-EGFP genome to obtain vAcCNE-KO-REP-EGFP. The arrowheads marks the transcription start sites (TSSs), the capital letters on a purple background – the first nucleotide to be transcribed. A rhombus marks the polyadenylation signal (PAS), a capital letter on yellow background – the last nucleotide to be transcribed. (PDF) [file pone.0095322.s004.pdf]
