# Supplementary material for: Identification of a Conserved Non-Protein-Coding Genomic Element that Plays an Essential Role in Alphabaculovirus Pathogenesis
Source: PLoS One. 2014 Apr 16;9(4):e95322. doi: 10.1371/journal.pone.0095322 (PMC3989284; doi:10.1371/journal.pone.0095322)
Supplement: Figure S2 — The HindIII-BglII genome fragment of the Malacosoma neustria nucleopolyhedrovirus (ManeMNPV). The letters on a green background identify the hoar sequence, on a turquoise background – the ManeNPV-specific ORFs. The start codons are underlined. The CNE sequence is indicated by the red bold letters. The letters on the blue, red, yellow, grey backgrounds mark HindIII, KpnI, PstI, BglII sites respectively. (PDF) [file pone.0095322.s002.pdf]

Figure S2

1 aagctttgtt gaacgacaaa attgtatcga gtggtagtgt gtgtgcatgt gtatacataa
61 atgtacttgt gttgtctaaa atgtcatctt ctaaagaatg cataaactct attaaataat
121 ctaacggctc accattcgaa acctcaggaa gagtcacggt gtcgtctaaa gatggttgac
181 tctcctcctg ttgacgctcc tgctgatgct gatcttgtcg atgctgattt tgctcttgct
241 gaatctgttc ctcttgatgt tcctgctgaa tctgttgaac ttgttcttgc tgaagaaaac
301 cgttcgctga cgtgctaggt acgtcgacgg ctgtagcgac agcgcttgtt atgagtagag
361 aatcttgaat ttcggtaaaa gacatgtcag agttttgaat cgttctcgtt taataagaag
421 gtatatgacg aggaccagta tcaccctcag acataggttt gacaagtggg gcttttctat
481 catccacaga aaaatattga ttatttacat cattaatcca attatcaata cgtctcaatg
541 catcatcggc cggtgttggt tagtcgattt gcagaagttt ctgcttcatc gccaatagtc
601 aatgcttgta tacttgaccc tacaatattt tcgtcgtcgt ggcgttcagt tgcattgcta
661 gtcgacatac attgactggt agaggcgata ggttctaaga cggtaccttg agtaaatccg
721 ctgccactag ctttgtttct ggcgtcggct ttgctgattt tactcacgct gttgattctg
781 ctggctacgc tgctaagtct gctgttcgtc ctattactga ccctggtgct ggcaataagg
841 cttgccctgg tgcgggccct agtgctggca ataaggcttg ctctggtgct tgccctggtg
901 ctggccctgg tgcttgccct ggtgcttacc gtggtgctgg ctgattgaaa agttttcata
961 ttgctgcttg aataaactgc taataaaatt gctcgaaccg aacatggcaa tgaaaggttt
1021 gttggccatt atatattaaa ttctaatcta aataatttag attatcacac cgatatacaa
1081 attttatcga attaaaccct tttcgggtaa ttgatgtgtt tactgtaaac ctgcagatag
1141 gaatagataa cga**actttttt tgcagtgcaa aattgtgcca tatttcgtag tgtatcgaga**
1201 gcatattgta cagtgtagac tatgtggcag catagtcttc gattcgtaga ttagatactg
1261 tatatgggtg ccgataaatt cttgaacttt tttgcagtac aaaaatttcc atgacgcatt
1321 ttgcgcacga caccatttgt acttatcaag gccgcagccg caccacgcgt gatcatgtga
1381 ccgtgctatc gatcacgcca ttcagttatc atatcacatg acaaggcagg atgtttgttg
1441 aagcggaaac ttttatcaag gtcgtcgcgt cggacatagg ggcgccgagt cttatcacat
1501 ccatgacgcg cgcgcacata catacacaca tcgacacttt acgaagtcta taaaaagatt
1561 gatcgcaatg tttgctta**ca gt**ttcattac gaaaacgcca cggtcaagat gagttctgaa
1621 tatcataaaa atattcaatt gtggtacaat ggtgttgaga atcaattggt ggtctatttg
1681 tgtttacccc aaacgaataa acgtgttcgt gttggttatt ggtgccagga gtccgacgag
1741 atcaaattca acgggatttc atttaaacat tacacccagt acaacagcaa aatggaggta
1801 tactataact atatatgtag tttaacgaat cctctgaaaa gaaagcatga agctattttg
1861 acgctgattg tcaaattaaa cgttatagag cacatcaatg aatatctaaa gttatatatg
1921 gagttctata gcaaagagag aactaatgta cccaagtata tgatgatgat caacgaaatg
1981 cttcaaagcg aaacactaga ttcgagcatg gagatagctg caaaagttta caaacgattc
2041 aataaactgt ccaaggaatt aagaagtgtt aacaccattg aaaagataga tct
