# Supplementary material for: Identification of a Conserved Non-Protein-Coding Genomic Element that Plays an Essential Role in Alphabaculovirus Pathogenesis
Source: PLoS One. 2014 Apr 16;9(4):e95322. doi: 10.1371/journal.pone.0095322 (PMC3989284; doi:10.1371/journal.pone.0095322)
Supplement: Figure S3 — Multiple CNE alignment. Consensus sequence is shown at the bottom of the alignment. The uppercase letters denote the completely conserved nucleotides, and the lowercase letters denote nucleotides with 50% or greater conservation. The letter “n” indicates that no completely or highly conserved nucleotides were found at the corresponding position. The points mark the positions in which the majority of the aligned sequences contain a gap. (PDF) [file pone.0095322.s003.pdf]

Figure S3

```
Apci AG-TTTTTTGCTGTGCAAAAATGTGACTTAAATCGTAGTATATTGAGAGCATATAGTACAGTGAAGACTATGTAAG-TAAAATAGTCTACGAATCG-AAATTTTCCACA-GGATATGGGCGAGTCATAATTAGTGACCTTTTTTGTACTGCAAAAAAAT155
Ro   AC-TTTTTTGTAATGCAAAAAAGTTGATAGTGTAGTAGTATATTGGGAGCGTATTGTACAGTGTAGACTATTCTAA-TAAAATAGTCTACGATTTGTAGAGTTTGTATT-GTATATTGAATGCCGAGCAAAAGTGAACTTTTTTGCATTGCAAAAAAGT156
Plxy AC-TTTTTTGTAATGCAAAAAAGTTGATAGTGTAGTAGTATATTGGGAGCGTATCGTACAGTGTAGACTATTCTAA-TAAAATAGTCTACGATTTGTAGAGATTGTACT-GTATATGGAGTGTCAGGCAAAAGTGAACTTTTTTGCATTGCAAAAAAAT156
Anpe AC-TTTTTTGCACTGCAAAATAGTTGGTTTTAGGGTAGTGTATTGGCAGCGTATAGAACAGTGTAGACTATTTAGG-TAAAATAGTCTACGAAACGTAGACTTTGTACT-GTATATGGGGCCGTGCGCAAAAACGTGTTTTTTTGTACTACAAAAAAGT156
Hycu AT-TTTTTTGCACTGCAAAAAAGTTTACTCTGTGGTAGTGTATTAGGAGCGTATAGAACAGTGTAGACTATTCAAG-TAAAATAGTCTACGAAATGTAGACTTTGCTCT-GTATATGGGGCTGTGTTCGATAACGTGTTTTTTTGCACTGCAAAAAAGT156
Ac   AC-TTTTTTGTAATGCAAAAAAGTTGATAGTGTAGTAGTATATTGGGAGCGTATCGTACAGTGTAGACTATTCTAA-TAAAATAGTCTACGATTTGTAGAGATTGTACT-GTATATGGAGTGTCAGGCAAAAGTGAACTTTTTTGCATTGCAAAAAAAT156
Orle ACTTTTTTTGTAGTGCAAAAAAGTGCTTTAAATCGTAGTATATTGAGAGCATATAGTACAGTGTAGACTATTCTAG-TAAAATAGTCTACGATTCGTAATATTG-TTCG-GTATATGCCCTGATATCAAACTCGATTTTTTTTGCACTACAAAAAAGT156
Boma AC-TTTTTTGTAATGCAAAAAAGTTGATAGTGTAGTAGTATATTGAGAGCGTATAGTACAGTGTAGACTATTCTAA-TAAAATAGTCTACGATTTGTAGAGTTCGTACT-GTATATTATATGTCAAACATAGGCGAACTTTTTTGTATTGCAAAAAAAT156
Mavi AC-TTTTTTGTAATGCAAAAAAGTTGATTGTGTAGTAGTATATTGGGAGCATATAGAACAGTGTAGACTATTCTAA-TAAAATAGTCTACGATTTGTAGACTTTGTACT-GTATATAGGGGTGCTGGCGAACGTGACTTTTTTTGCATTACAAAAAAAT156
Bm   AC-TTTTTTGTAATGCAAAAAAGTTGATAGTGTAGTAGTATATTGAGAGCGTATAGTACGTGTAGACTATTCTAA-TAAAATAGTCTACGATTTGTAGAGTTCGTACT-GTATATTATATGTCAAACATAGGCGAACTTTTTTGTATTGCAAAAAAATA156
Ld   AC-TTTTTTGCACTGCAAAAAAGCAGAGGAAATCGTAGTGTATGGAGAGCGTCTTCTACATCGTAGACTATG-CGG-GTCAATAGTCTACGATCG-CAGCGTTTCCTCT-GTATATGGGTCCTGGATTCGGAGGCCACTTTTTTGCAGTGCAAAAAAAG154
Lyxy AC-TTTTTTGCAGTGCAAAAAAGTAAAGGAAATCGTAGTGTATGGAGAGCATCTTCTACATCGTAGACTATG-CGG-TTCAATAGTCTACGATTG-AACCGTTTCCTCT-GTATATGGGTGTCAAATTTAGAGGCTCTTTTTTTGCACTGCAAAAAAGT154
Clbi AC-TTTTTTGCACTTCAAAAAAAACATTATAATTATAGTATATTGGGAGCATATCTTACACTGTAGACTATGTAAA-ATAAATAGTCTACGATTT-GAAATAATGTTCT-GTATATTGAAGCGTGAGATATAGCGACTTTTTTTGCATTACAAAAAAGT155
Agip AC-TTTTTTGTAGTGCAAAAATGTACCTTAAAGTGTAGTATATGGAGAGCATATTGTACAGTGTAGACTATGCTAG-TATAATAGTCTACGATTCG-AAATTTTCCACT-GTATATCGAGGTCATAATTCTAATCAATATTTTTGCACTCCAAAAAAGG154
Splt║AC-TTTTTTGCAGTGCAAAAATGTGCCCTAAAGTGTAGTATATGGAGAGCATATTGTTCAGTGTAGACTATGCTAG-TATAATAGTCTACGATTCG-AAATTTTCCACT-GTATATCGGTCTTTGTATGGAACCTAATTTTTTTGTACTGCAAAAAAGT155
Sf   AC-TTTTTTGCAGTGCAAAAAAGTACCTTAAAGTGTAGTATATGGAGAGCATATTCTACAGTGTAGACTATGCTAG-TATAATAGTCTACGATTCG-AACTATTGTTCT-GTATATAGATATGCCATATCTAATCAACATTTTTGCACTACAAAAAAGT155
MacoAAC-TTTTTTGCAGTGCAAAAAAAGTCTCTATAGTGTAGTATATAGGGCCCATATACTACAGTGTAGACTATGCTAG-TATAATAGTCTACGATTCG-AAATATTCCACT-GTATATCGGTGTAGGATCAGAGGTGACTTTTTTTGCACTGCAAAAAAGT155
Ecob ACTTTTTTTGCACTGCAAAAAATGACGCAATAATGTAATATATTGCCAGCATATAGTACAGTGTAGACTATGCTAG-TAAAATAGTCTACGAATCG-AATTTTTGCTCT-GTATATAGGGGCAGTCTTTAAACCCAATTTTTTTGTTGTGCAAAAAAAG156
Splt AC-TTTTTTGCAGTGCAAAAATATACTCTATTTCGTAGTATATAGAATCGATATA-TACAGTGTAGACTTTGCTAG-ATAAATAGTCTACAATTCG-AATTTTTGTACT-GTATATAGCATCCTGCCGATAGCCGAATTTTTTTGCACTGCAAAAAAAA154
HearSAC-ATTTTTGTTGTGCAAAAATGTACCTATTTTTGTAGTATATTGGGAGCATATCGTACAGTGTAGACTATTCTGG-TTAAATAGTCTTCGATTCG-AAAATTTCCACT-GTATATTGATGACGTCATTAACACGAATTTTTTTGTAGTGCAAAAAAAT155
Lese ACTTTTTTTGCAGTGCAAAAATGTGCTCTGTGTCGTAGTATATAGGTAGCATATCGTACAGTGTAGACTTTGCTAG-TTAAAAAGTCTTCGAATCG-AACTATTCCACT-GTATATAGGCTCTGATTTTTTATCGTCTTTTTTTGCACTACAAAAAAGT156
HearMAC-TTTTTTGCAGTGCAAAAAAAGTCGTTATAGTGTAGTATATAGGAACGATATCGTACAGTGTAGACTATGCTAG-TATAATAGTCTACGATTCG-AAATATTCCACT-GTATATTGGTGTCGGAATAGAGGTGACTTTTTTTGCAGTGCAAAAAAGT155
Se   AC-TTTTTTGCACTGCAAAAATGCACCTTAAAGTGTAGTATATGGAGAGCATATTGTTCAGTGTAGACTATGCTAG-TATAATAGTCTACGATTCG-AAATTTTCCATT-GTATATCGGCCTTGGAATGATGACCAACTTTTTTGCACTACAAAAAAGT155
Mb   AC-TTTTTTGCAGTGCAAAAAAAGTCGTTATAGTGTAGTATATAGGAACGATATCGTACAGTGTAGACTATGCTAG-TATAATAGTCTACGATTCA-AAATATTCCACT-GTATATTGGTGTCGGATTAGAGGTGACTTTTTTTGCAGTGCAAAAAAGT155
MacoBAC-TTTTTTGCAGTGCAAAAAAAGTCGTTATAGTGTAGTATATAGGAACGATATCGTACAGTGTAGACTATGCTAG-TATAATAGTCTACGATTCG-AAATATTCCATT-GTATATTGGTGTCGGATTAGAGGTGACTTTTTTTGCAGTGCAAAAAAGT155
Agse AT-TTTTTTGCTGTGCAAAAAAGTGCCTTAAAGTGTAGTATATGGAGAGGATATATTACAGTGTAGACTATGCTAG-TATAATAGTCTACGATTCGAACTTTTCCACT--GTATATTGGCCTTGGAAAAATGATGACTATTTTTGCACTGCAAAAATGT155
Eups AG-TTTTTTGTTATACAAAAAACCTCCTAAAAATGTAGTATATAGAGAGCATATAATGCAGTGTAGACTATTCAGG-GTAAATAGTCTACGATTTGTAATTTTCCGCT--GTATATTTGACCTTGAAAATAAAGCAATTTTTTTGCACTACAAAAAAGT155
Ag   A-CTTTATTGCAGTGCAAAAAAGTTCAATGTGCGGTAGTGTATTGGGAGCGTATACGACAGTGTAGACTATTCAAG-TTAAATAGTCTACGAAACGTAGAGTTTGTATGTGTATATAGGGTATAGGACAAATGTGAATTTTTTTGCATTGCAATAAAGT157
CfDEFAC-TTTATTGCAGTGCAAAAAAGTTCGATGTGTGGTAGTGTATTGGGAGCGTATACAACAGTGTAGACTATTCAAG-TTAAATAGTCTACGAAACGTAGAGTTCGTATGTGTATATAGGGTGCGATATAAAAGTGAACTTTTTTGCACTGCAATAATGT157
Op   AC-TTTTTTGCACTGCAAAAAGGTTCAATGTGTGGTAGTGTATTTGGAGCGTATACAACGGTGTAGACTATTTATG-TAAAATAGTCTACGAAACGTAGAGTTTGTAC-TATGTATGGGCCCGCGTGCAAAAGCGTGTTTTTTTGCAGTGCAAAAAAGT156
Cf   AC-TTTTTTGCAGTGCAAAAATGTACATTACGTGGTAGTGTATAGAGAGCATATTAAACAGTGTAGACTATTAAAG-TAAAATAGTCTACGAAACGAAGACTTTGTTC-TGTATATGG-GTCCTTGAAAATAATGATTATTTTTGCACTTCAAAAAAGT155
Thor GC-TTTTTTGCATTGCAAAAAAGTTGATTATGTGGTAGTATATTAGGAGCGTATTGTACAGTGTAGACTATTCTAA-TAAAATAGTCTACGATTTGTAGAGTTTGTAC-TGTATATAGGGTGCCAGCCAAAATTAAACTTTTTTGAATTGCAAAAAAAT156
Eppo AA-TTTTTTGCACTACAAAAAAATTCAATGTGTAGTAGTGTATTAGGAGCGTATAGAACAGTGTAGACTATTCAAG-TTAAATAGTCTACGATTCGTAGTTTTTGTAC-TGTGTATAGGGTCTCAGAAAAAAATGAACTTTTTTGCAGTGCAAAAAGGT156
Mane AC-TTTTTTGCAGTGCAAAATTGTGCCATATTTCGTAGTGTATCGAGAGCATATTGTACAGTGTAGACTATGTGG--CAGCATAGTCTTCGATTCGTAGATTAGATAC-TGTATATGGGTGCCGATAAATTCTTGAACTTTTTTGCAGTACAAAAATTT155
Adho AC-TTTTTTGTCCTTCAAAAAAGGACCTTAAAGTGTAGTGTATTTAGAGCATATAGTACAGTGTAGACTTTGTCTATTAAAACAGTCTACGATTCG-AAATTTCGTTCG-GGATATGGGTTGGAAAAAATTAGGTTACTTTTTTGTAGTGCAAAAAAAT156
Ador AC-TTTTTTGTCCTTCAAAAAAGGACCTTAAAGTGTAGTGTATTTAGAGCATATAGTACAGTGTAGACTTTGTCTATTAAAACAGTCTACGATTTG-AAATTTTGTTCT-GGATATGAGTAAAAAAAAATTAGGGTACTTTTTTGCACTGCAAAAAAAT156
Cc   AC-TTTTTTGTCGTGCAAAAAAGTACTCTAAAGTGTAGTATATTTACAGCATATTGTACAGTGTAGACTAT-TCTAGTTAAATAGTCTACGATTAGTAGATTTTGTTCT-GTATATTGATGCCGATACGATAACGAACTTTTTTGTAGTGCAAAAAAAT156
Tn   AC-TTTTTTGTTGTGCAAAAAAGGACTCAAAAATGTAGTATATTGACAGCATATTGTTCAGTGTAGACTAT-TCTAGTTAAATAGTCTACGATTAGTAGTTTTTGTACT-GTATATTGATGCCGCCCCGATAACGAACTTTTTTGCACTACAAAAAAAG156
cons ac.tTTtTTGcagTgCAAAAaagtgcattatatngTAgTaTATtgagagcaTaTngtaCagtGtAGACTaTgctag.taaaAtAGTCTaCgAttcgnAaattttgtact.gtaTATgggnnnnagagnaaaagngaattTTTTTGcanTgCAAaAAagt
```
