# Supplementary material for: Identification of a Conserved Non-Protein-Coding Genomic Element that Plays an Essential Role in Alphabaculovirus Pathogenesis
Source: PLoS One. 2014 Apr 16;9(4):e95322. doi: 10.1371/journal.pone.0095322 (PMC3989284; doi:10.1371/journal.pone.0095322)
Supplement: Figure S5 — The 200-bp CNE-containing sequences of 38 alphabaculovirus genomes. The letters on a gray background marks the left, central and right dyad symmetry elements (DSl, DSc, DSr respectively). The Dsc, DSl, and DSr are extracted from each CNE to demonstrate the extent of their IRs with the respect to the core IRs (core IRs are underlined). (PDF) [file pone.0095322.s005.pdf]

Figure S5

1. Adoxophyes honmai nucleopolyhedrovirus
8334-8534
NC_004690

CCACCACAGCTTGCCCTATGTATGAA**CTTTTTTGTCCTTCAAAAAAG**GACCTTAAAGTGTAGTGT
ATTTAGAGCATATAGTACAGT**GTAGACTTTGTCTATTAAAACAGTCTAC**GATTCGAAATTTCGTT
CGGGATATGGGTTGGAAAAAATTAGGTTAC**TTTTTTGTAGTGCAAAAAA**ATGAAAATTGAAAAT
AGATATA

GTAGACTTTGTCTATTAAAACAGTCTAC-DSc

CTTTTTTGTCCTTCAAAAAAG-DSl   **TTTTTTGTAGTGCAAAAAA-DSr**

---

2. Adoxophyes orana nucleopolyhedrovirus
8141-8340
NC_011423

CACCACAGCTTGCCTATGTATGAA**CTTTTTTGTCCTTCAAAAAAG**GACCTTAAAGTGTAGTGTATT
TAGAGCATATAGTACAGT**GTAGACTTTGTCTATTAAAACAGTCTAC**GATTTGAAATTTTGTTCTG
GATATGAGTAAAAAAAAATTAGGGTAC**TTTTTTGCACTGCAAAAAA**ATGAAATTAAAAAATTTAT
ATAG

GTAGACTTTGTCTATTAAAACAGTCTAC-DSc

CTTTTTTGTCCTTCAAAAAAG-DSl   **TTTTTTGCACTGCAAAAAA-DSr**

---

3. Agrotis ipsilon multiple nucleopolyhedrovirus strain Illinois
6127-6326
NC_011345

CGCCTATGCACTTTTGATAGTGAACT**TTTTTGTAGTGCAAAAA**TGTACCTTAAAGTGTAGTATATG
GAGAGCATATTGTACAGT**GTAGACTATGCTAGTATAATAGTCTAC**GATTCGAAATTTTCCACTGT
ATATCGAGGTCATAATTCTAATCAATA**TTTTTGCACTCCAAAAA**AGGCGATAGCGGAAACGAGGC
AGAG

GTAGACTATGCTAGTATAATAGTCTAC-DSc

TTTTTGTAGTGCAAAAA-DSl   **TTTTTGCACTCCAAAAA-DSr**

---

4. Agrotis segetum nucleopolyhedrovirus
5898-6098
NC_007921

AGGTCGTCACGGTTAGTCACGCAGCCAT**TTTTTTGCTGTGCAAAAAA**GTGCCTTAAAGTGTAGT
ATATGGAGAGGATATATTACAGT**GTAGACTATGCTAGTATAATAGTCTAC**GATTCGAACTTTT
CCACTGTATATTGGCCTTGGAAAAATGATGACT**ATTTTTGCACTGCAAAAAT**GTTGATAAGCCT
CAGGAGGTGG

GTAGACTATGCTAGTATAATAGTCTAC-DSc

TTTTTTGCTGTGCAAAAAA-DSl **ATTTTTGCACTGCAAAAAT-DSr**

---

5. Antheraea pernyi multiple nucleopolyhedrovirus-L2
6950-7150
NC_008035

TTGCAGTACGTGAGTAAAGTAACGTACTT**TTTTGCACTGCAAAA**TAGTTGGTTTTAGGGTAGTG
TATTGGCAGCGTATAGAACAGT**GTAGACTATTTAGGTAAAATAGTCTAC**GAAACGTAGACTTT
GTACTGTATATGGGGCCGTGCGCAAAAACGTGT**TTTTTTGTACTACAAAAAA**GTTGGCGCAAAT
TTATTAATTA

GTAGACTATTTAGGTAAAATAGTCTAC-DSc

TTTTGCACTGCAAAA -DSl **TTTTTTGTACTACAAAAAA-DSr**

---

6. Anticarsia gemmatalis multiple nucleopolyhedrovirus, strain2D
(126100-126300)
NC_008520

TTATAATTTAATA**TGAACTTTATTGCAGTGCAAAAAAGTTCA**ATGTGCGGTAGTGTATTGGGA
GCGTATACGACAGT**GTAGACTATTCAAGTTAAATAGTCTAC**GAAACGTAGAGTTTGTATGTGT
ATATAGGGTATAGGACAAATG**TGAATTTTTTTGCATTGCAATAAAGTTCA**TATCTGCAAGTAA
CGTGTTTACGTG

GTAGACTATTCAAGTTAAATAGTCTAC-DSc

TGAACTTTATTGCAGTGCAAAAAAGTTCA -DSl **TGAATTTTTTTGCATTGCAATAAAGTTCA-DSr**

---

7. Apocheima cinerarium nucleopolyhedrovirus
(118818-118619)
FJ914221

TAAATAATGATATCATTGATTGTAAGT**TTTTTGCTGTGCAAAAA**TGTGACTTAAATCGTAGTAT
ATTGAGAGCATATAGTACAGT**GAAGACTATGTAAGTAAAATAGTCTAC**GAATCGAAATTTTCC
ACAGGATATGGGCGAGTCATAATTAGTGACC**TTTTTTGTACTGCAAAAAA**ATTCATATCTGATA
TGGTGATCAA

GAAGACTATGTAAGTAAAATAGTCTAC-DSc

TTTTTGCTGTGCAAAAA-DSl **TTTTTTGTACTGCAAAAAA-DSr**

---

8. Autographa californica multiple nucleopolyhedrovirus clone 6, complete genome

132204-132404
NC_001623

GGGGAACTTGCCAGGCAAAAATG**AACTTTTTTGTAATGCAAAAAAGTT**GATAGTGTAGTAGTA
TATTGGGAGCGTATCGTACAGT**GTAGACTATTCTAATAAAATAGTCTAC**GATTTGTAGAGATTG
TACTGTATATGGAGTGTCAGGCAAAAGTGAAC**TTTTTTGCATTGCAAAAAA**ATTCTTTTAAATT
TATCATATC

GTAGACTATTCTAATAAAATAGTCTAC-DSc

AACTTTTTTGTAATGCAAAAAAGTT-DSl **TTTTTTGCATTGCAAAAAA-DSr**

---

9. Bombyx mandarina nucleopolyhedrovirus, isolate S1
121284-121483
NC_012672

GGGAACTTGTCAGGCAAAAATG**AACTTTTTTGTAATGCAAAAAAGTT**GATAGTGTAGTAGTAT
ATTGAGAGCGTATAGTACAGT**GTAGACTATTCTAATAAAATAGTCTAC**GATTTGTAGAGTTCGT
ACTGTATATTATATGTCAAACATAGGCGAAC**TTTTTTGTATTGCAAAAAA**ATTCATTTTTTAATT
TATCATAT

GTAGACTATTCTAATAAAATAGTCTAC-DSc

AACTTTTTTGTAATGCAAAAAAGTT-DSl **TTTTTTGTATTGCAAAAAA-DSr**

---

10. Bombyx mori nuclear polyhedrosis virus, isolate T3
122051-122251
NC_001962

AGGGAACTTGTCAGGCAAAAATG**AACTTTTTTGTAATGCAAAAAAGTT**GATAGTGTAGTAGTA
TATTGAGAGCGTATAGTACAGT**GTAGACTATTCTAATAAAATAGTCTAC**GATTTGTAGAGTTCG
TACTGTATATTATATGTCAAACATAGGCGAAC**TTTTTTGTATTGCAAAAAA**ATTCATTTTTTAAT
TTATCATAT

GTAGACTATTCTAATAAAATAGTCTAC-DSc

AACTTTTTTGTAATGCAAAAAAGTT-DSl **TTTTTTGTATTGCAAAAAA-DSr**

---

11. Choristoneura fumiferana MNPV
124695-124895
NC 004778

GCCGAGTCACGTAGGCGCGCGCGCAACT**TTTTTGCAGTGCAAAAA**TGTACATTACGTGGTAGTG
TATAGAGAGCATATTAAACAGT**GTAGACTATTAAAGTAAAATAGTCTAC**GAAACGAAGACTTT
GTTCTGTATATGGGTCCTTGAAAATAATGATTA**TTTTTGCACTTCAAAAA**AGTTCATAACAACA
CGGAACTAAA

GTAGACTATTAAAGTAAAATAGTCTAC-DSc

TTTTTGCAGTGCAAAAA-DSl **TTTTTGCACTTCAAAAA-DSr**

---

12. Choristoneura fumiferana defective multiple nucleopolyhedrovirus
(125520-125720)
NC_005137

TGTACGCAAACATGTTAAGAGT**GAACTTTATTGCAGTGCAAAAAAGTTC**GATGTGTGGTAGTG
TATTGGGAGCGTATACAACAGT**GTAGACTATTCAAGTTAAATAGTCTAC**GAAACGTAGAGTTC
GTATGTGTATATAGGGTGCGATATAAAAG**TGAACTTTTTTGCACTGCAATAATGTTCA**TATTA
GCCTATCACATG

GTAGACTATTCAAGTTAAATAGTCTAC-DSc

GAACTTTATTGCAGTGCAAAAAAGTTC-DSl **TGAACTTTTTTGCACTGCAATAATGTTCA-DSr**

---

13. Chrysodeixis chalcites nucleopolyhedrovirus
6632-6832
NC_007151

CATAAATATGATATAGCTCGAGATAA**ACTTTTTTGTCGTGCAAAAAAGT**ACTCTAAAGTGTAG
TATATTTACAGCATATTGTACAGT**GTAGACTATTCTAGTTAAATAGTCTAC**GATTAGTAGATTT
TGTTCTGTATATTGATGCCGATACGATAACGAAC**TTTTTTGTAGTGCAAAAAA**ATACGATAACG
ATTATCGATA

GTAGACTATTCTAGTTAAATAGTCTAC-DSc

ACTTTTTTGTCGTGCAAAAAAGT-DSl **TTTTTTGTAGTGCAAAAAA-DSr**

---

14. Clanis bilineata nucleopolyhedrosis virus, strain DZ1
(6263-6463)
NC_008293

GTTTAATTTGTTAATGAAC**TTTTTTGCACTTCAAAAAA**AACATTATAATTATAGTATATTGGGA
GCATATCTTA**CACTGTAGACTATGTAAAATAAATAGTCTAC**GATTTGAAATAATGTTCTGTATA
TTGAAGCGTGAGATATAGCGACT**TTTTTTGCATTACAAAAAA**GTTCATATAATTTTATGTGTA
AAAATAATA

GTAGACTATGTAAAATAAATAGTCTAC-DSc

TTTTTTGCACTTCAAAAAA-DSl **TTTTTTGCATTACAAAAAA-DSr**

---

15. Ecotropis obliqua NPV strain A1
6464-6664
NC_008586

CGCTCTATACGTACATGAGGTCACCACT**TTTTTTGCACTGCAAAAAA**TGACGCAATAATGTAAT
ATATTGCCAGCATATAGTACAGT**GTAGACTATGCTAGTAAAATAGTCTAC**GAATCGAATTTTT
GCTCTGTATATAGGGGCAGTCTTTAAACCCAA**TTTTTTTGTTGTGCAAAAAAA**GACGAGATAAG
ATTGTAAGCT

GTAGACTATGCTAGTAAAATAGTCTAC-DSc

TTTTTTGCACTGCAAAAAA-DSl **TTTTTTTGTTGTGCAAAAAAA-DSr**

---

16. Epiphyas postvittana nucleopolyhedrovirus
114611-114811
NC_003083

CAAACACACTCACACGTAAATATGTAA**TTTTTTGCACTACAAAAAA**ATTCAATGTGTAGTAGTG
TATTAGGAGCGTATAGAACAGT**GTAGACTATTCAAGTTAAATAGTCTAC**GATTCGTAGTTTTTG
TACTGTGTATAGGGTCTCAGAAAAAAATGAACT**TTTTTGCAGTGCAAAAA**GGTTCAGTATTGCT
TTACAGTAA

GTAGACTATTCAAGTTAAATAGTCTAC-DSc

TTTTTTGCACTACAAAAAA-DSl **TTTTTGCAGTGCAAAAA-DSr**

---

17. Euproctis pseudoconspersa nucleopolyhedrovirus strain Hangzhou
5220-5419 plus
NC_012639

TTATCCTTTGTTTTATACAGTA**GTTTTTTGTTATACAAAAAAC**CTCCTAAAAATGTAGTATATAG
AGAGCATATAATGCAGT**GTAGACTATTCAGGGTAAATAGTCTAC**GATTTGTAATTTTCCGCTGT
ATATTTGACCTTGAAAATAAAGCAAT**TTTTTTGCACTACAAAAAA**GTACGTGTTCAAATTGTAT
GTGTGTG

GTAGACTATTCAGGGTAAATAGTCTAC-DSc

GTTTTTTGTTATACAAAAAAC-DSl **TTTTTTGCACTACAAAAAA-DSr**

---

18. Helicoverpa armigera multiple nucleopolyhedrovirus
9210-9310
NC_011615

ATCGGATTAGGCGATAACAAAC**TTTTTTGCAGTGCAAAAAA**AGTCGTTATAGTGTAGTATATA
GGAACGATATCGTACAGT**GTAGACTATGCTAGTATAATAGTCTAC**GATTCGAAATATTCCAC
TGTATATTGGTGTCGGAATAGAGGTGACT**TTTTTTGCAGTGCAAAAAA**GTACGTGACAAAAGC
CACGTACAATATA

GTAGACTATGCTAGTATAATAGTCTAC-DSc

TTTTTTGCAGTGCAAAAAA-DSl **TTTTTTGCAGTGCAAAAAA-DSr**

---

19. Helicoverpa armigera nuclear polyhedrosis virus, strainC1
5398-5598
NC_003094

GACGTGATAGAGATAACGTATCAC**ACATTTTTGTTGTGCAAAAATGT**ACCTATTTTTGTAGTA
TATTGGGAGCATATCGTACAGT**GTAGACTATTCTGGTTAAATAGTCTTC**GATTCGAAAATTTC
CACTGTATATTGATGACGTCATTAACAC**GAATTTTTTTGTAGTGCAAAAAAATTC**AGGTCGCT
TCGACAACACTT

GTAGACTATTCTGGTTAAATAGTCTTC-DSc

ACATTTTTGTTGTGCAAAAATGT-DSl **GAATTTTTTTGTAGTGCAAAAAAATTC-DSr**

---

20. Hyphantria cunea nucleopolyhedrovirus
7113-7317
NC_007767

GCGGAGTACGTGACTCTCGACACGTAT**TTTTTTGCACTGCAAAAAA**GTTTACTCTGTGGTA
GTGTATTAGGAGCGTATAGAACAGT**GTAGACTATTCAAGTAAAATAGTCTAC**GAAATGTA
GACTTTGCTCTGTATATGGGGCTGTGTTCGATAACGTGT**TTTTTTGCACTGCAAAAAA**GTTC
ATGTGTGACAAGCAAGATTATC

GTAGACTATTCAAGTAAAATAGTCTAC-DSc

TTTTTTGCACTGCAAAAAA-DSl **TTTTTTGCACTGCAAAAAA-DSr**

---

21. Leucania separata nuclear polyhedrosis virus strain AH1
8462-8662
NC_008348

GCTCATGGGCTGCAATCGAGCTCGACTT**TTTTTGCAGTGCAAAAA**TGTGCTCTGTGTCGTAG
TATATAGGTAGCATATCGTACAGT**GTAGACTTTGCTAGTTAAAAAGTCTTC**GAATCGAACT
ATTCCACTGTATATAGGCTCTGATTTTTTATCGTCT**TTTTTTGCACTACAAAAAA**GTTCAATC
ATCGTTGTGACGTCA

GTAGACTTTGCTAGTTAAAAAGTCTTC-DSc

TTTTTGCAGTGCAAAAA-DSl **TTTTTTGCACTACAAAAAA-DSr**

---

22. Lymantria dispar nucleopolyhedrovirus
10008-10208
NC_001973

CCGGCTCCGGCCAGGCCCGGACAGA**CTTTTTTGCACTGCAAAAAAG**CAGAGGAAATCGTA
GTGTATGGAGAGCGTCTTCTAC**ATCGTAGACTATGCGGGTCAATAGTCTACGAT**CGCAGC
GTTTCCTCTGTATATGGGTCCTGGATTCGGAGGCCAC**TTTTTTGCAGTGCAAAAAA**AGTGG
GTCTGCGCCGCCGGTCCGCC

ATCGTAGACTATGCGGGTCAATAGTCTACGAT-DSc

CTTTTTTGCACTGCAAAAAAG-DSl **TTTTTTGCAGTGCAAAAAA-DSr**

---

23. Lymantria xylina MNPV isolate LyxyMNPV-5
8208-8407
NC_013953

AGCTCCGGCCAGGCGCAAACAG**ACTTTTTTGCAGTGCAAAAAAGT**AAAGGAAATCGTAGTG
TATGGAGAGCATCTTCTAC**ATCGTAGACTATGCGGTTCAATAGTCTACGAT**TGAACCGTTT
CCTCTGTATATGGGTGTCAAATTTAGAGGCTCT**TTTTTTGCACTGCAAAAAA**GTTAATTCCTA
CCGACGCGTCCAAAC

ATCGTAGACTATGCGGTTCAATAGTCTACGAT-DSc

ACTTTTTTGCAGTGCAAAAAAGT-DSl **TTTTTTGCACTGCAAAAAA-DSr**

---

24. Malacosoma neustria NPV (Mane)

CCTGCAGATAGGAATAGATAACGAACTTTTTTGCAGTGCAAAATTGTGCCATATTTCGTAGTG
TATCGAGAGCATATTGTACAGTGTAGACTATGTGGCAGCATAGTCTTCGATTCGTAGATTAGA
TACTGTATATGGGTGCCGATAAATTCTTGAACTTTTTTGCAGTACAAAAATTTCCATGACGCAT
TTTGCGCGCA

GTAGACTATGTGGCAGCATAGTCTTC-DSc

TTTTGCAGTGCAAAA-DSl **TTTTTGCAGTACAAAAA-DSr**

---

25. Mamestrae brassicae MNPV
(9190-9390)
JQ798165

ATCGTTATCGGATTAGGCGATAACAAACTTTTTTGCAGTGCAAAAAAAGTCGTTATAGTGTA
GTATATAGGAACGATATCGTACAGTGTAGACTATGCTAGTATAATAGTCTACGATTCAAAA
TATTCCACTGTATATTGGTGTCGGATTAGAGGTGACTTTTTTTGCAGTGCAAAAAAGTACGT
GACAAAAGCCACGTAC

GTAGACTATGCTAGTATAATAGTCTAC-DSc

TTTTTTGCAGTGCAAAAAA-Dsl **TTTTTTGCAGTGCAAAAAA-DSr**

---

26. Mamestra configurata nucleopolyhedrovirus B
(9189-9389)
NC_004117

CGTTATCGGATTAGGCGATAACGAACTTTTTTGCAGTGCAAAAAAAGTCGTTATAGTGTAGTA
TATAGGAACGATATCGTACAGTGTAGACTATGCTAGTATAATAGTCTACGATTCGAAATATTC
CATTGTATATTGGTGTCGGATTAGAGGTGACTTTTTTTGCAGTGCAAAAAAGTACGTGACAAA
AGCCACGTACAA

GTAGACTATGCTAGTATAATAGTCTAC-DSc

TTTTTTGCAGTGCAAAAAA-DSl **TTTTTTGCAGTGCAAAAAA-DSr**

---

27. Mamestra configurata nucleopolyhedrovirus strain 90/2
(9010-9210)
NC_003529

CAGATTAGGCGATAACGATAATGAACTTTTTTGCAGTGCAAAAAAAGTCTCTATAGTGTAGT
ATATAGGGCCCATATACTACAGTGTAGACTATGCTAGTATAATAGTCTACGATTCGAAATAT
TCCACTGTATATCGGTGTAGGATCAGAGGTGACTTTTTTTGCACTGCAAAAAAGTACGTGCC
GTCGGTCACGTACAA

GTAGACTATGCTAGTATAATAGTCTAC-DSc

TTTTTTGCAGTGCAAAAAA-DSl **TTTTTTGCACTGCAAAAAA-DSr**

---

28. Maruca vitrata MNPV
107503-107703
NC_008725

GCAAGCAAAATTCAACTTTTTTGTAATGCAAAAAAGTTGATTGTGTAGTAGTATATTGGGA
GCATATAGAACAGTGTAGACTATTCTAATAAAATAGTCTACGATTTGTAGACTTTGTACTGT
ATATAGGGGTGCTGGCGAACGTGACTTTTTTTGCATTACAAAAAAATTCATATCTGATACGA
TCTGTTATTCAAGCGT

GTAGACTATTCTAATAAAATAGTCTAC-**DSc**

TCAACTTTTTTGTAATGCAAAAAAGTTGA-**DSl** TTTTTTTGCATTACAAAAAAA-**DSr**

---

29. Orgyia leucostigma NPV isolate CFS-77
5392-5591
NC_010276

GATTAGATATATTCGTATCGACTTTTTTTGTAGTGCAAAAAAGTGCTTTAAATCGTAGTATAT
TGAGAGCATATAGTACAGTGTAGACTATTCTAGTAAAATAGTCTACGATTCGTAATATTGTT
CGGTATATGGCCCTGATATCAAACTCGATTTTTTTTGCACTACAAAAAAGTTGACGTCTGATT
TTCGACTTTATT

GTAGACTATTCTAGTAAAATAGTCTAC-**DSc**

TTTTTTTGTAGTGCAAAAAAA-**DSl** TTTTTTTGCACTACAAAAAAA-**DSr**

---

30. Orgyia pseudotsugata multicapsid nucleopolyhedrovirus
(128266-128466)
NC_001875

GCCGGCCTACGTGACTGCCGACACGTACTTTTTTGCACTGCAAAAAGGTTCAATGTGTGGTA
GTGTATTTGGAGCGTATACAACGGTGTAGACTATTTATGTAAAATAGTCTACGAAACGTAG
AGTTTGTACTATGTATGGGCCCGCGTGCAAAAGCGTGTTTTTTTGCAGTGCAAAAAAGTTGG
TGGTGGGGAGGCCAC

GTAGACTATTTATGTAAAATAGTCTAC-**DSc**

TTTTTGCACTGCAAAAA-**DSl** TTTTTTGCAGTGCAAAAAA-**DSr**

---

31. Plutella xylostella multiple nucleopolyhedrovirus isolate CL3
132723-132923
NC_008349

GGTGGGGAACTTGCCAGACAAAAATGAACTTTTTTGTAATGCAAAAAAGTTGATAGTGTAG
TAGTATATTGGGAGCGTATCGTACAGTGTAGACTATTCTAATAAAATAGTCTACGATTTGTA
GAGATTGTACTGTATATGGAGTGTCAGGCAAAAGTGAACTTTTTTGCATTGCAAAAAAATTC
ATTTTAAATTTATCAT

GTAGACTATTCTAATAAAATAGTCTAC-**DSc**

AACTTTTTTGTAATGCAAAAAAGTT-**DSl** TTTTTTGCATTGCAAAAAA-**DSr**

---

32. Rachiplusia ou multiple nucleopolyhedrovirus
129821-130002
NC_004323

GGGGAACTTGCCAAGCAATAATGAACTTTTTTGTAATGCAAAAAAGTTGATAGTGTAGTAG
TATATTGGGAGCGTATTGTACAGTGTAGACTATTCTAATAAAATAGTCTACGATTTGTAGAG
TTTGTATTGTATATTGAATGCCGAGCAAAAGTGAACTTTTTTGCATTGCAAAAAAGTTCATT
TTAATTTTAAATTTTT

GTAGACTATTCTAATAAAATAGTCTAC-DSc

AACTTTTTTGTAATGCAAAAAAGTT-DSl **TGAACTTTTTTGCATTGCAAAAAAGTTCA-DSr**

---

33. Spodoptera exigua nucleopolyhedrovirus
5848-6048
NC_002169

TATCTTATCTGAAAATGATATTGAACTTTTTTGCACTGCAAAAATGCACCTTAAAGTGTAGTA
TATGGAGAGCATATTGTTCAGTGTAGACTATGCTAGTATAATAGTCTACGATTCGAAATTTT
CCATTGTATATCGGCCTTGGAATGATGACCAACTTTTTTGCACTACAAAAAAGTACGTGACC
GATAATCCAGATAA

GTAGACTATGCTAGTATAATAGTCTAC-DSc

TTTTTGCACTGCAAAAA-DSl **ACTTTTTTGCACTACAAAAAAGT-DSr**

---

34. Spodoptera frugiperda MNPV, strain 3AP2
5058-5258
NC_009011

TTAGGTTCAAGGCCTGTGCGTCATCAACTTTTTTGCAGTGCAAAAAAGTACCTTAAAGTGTAG
TATATGGAGAGCATATTCTACAGTGTAGACTATGCTAGTATAATAGTCTACGATTCGAACTAT
TGTTCTGTATATAGATATGCCATATCTAATCAACATTTTTGCACTACAAAAAAGTACACTACC
ATTTTTATCTTG

GTAGACTATGCTAGTATAATAGTCTAC-DSc

ACTTTTTTGCAGTGCAAAAAAGT-DSl **TTTTTGCACTACAAAAA-DSr**

---

35. Spodoptera litura nucleopolyhedrovirus strain G2
6006-6205
NC_003102

CGGTCACGTAGGCCGCCGATGTCAACTTTTTTGCAGTGCAAAAATATACTCTATTTCGTAGTA
TATAGAATCGATATATACAGTGTAGACTTTGCTAGATAAATAGTCTACAATTCGAATTTTTG
TACTGTATATAGCATCCTGCCGATAGCCGAATTTTTTTGCACTGCAAAAAAAATTGATATCT
AAATCCGGAGACG

TGTAGACTTTGCTAGATAAATAGTCTACA-DSc

TTTTTGCAGTGCAAAAA-DSl **TTTTTTTGCACTGCAAAAAAA-DSr**

---

36. Spodoptera litura nucleopolyhedrovirus II
6034-6233
NC_011616

CGATGGCCACGTGCAGGCGATAACCAACT**TTTTTGCAGTGCAAAAA**TGTGCCCTAAAGTGTA
GTATATGGAGAGCATATTGTTCAGT**GTAGACTATGCTAGTATAATAGTCTAC**GATTCGAAAT
TTTCCACTGTATATCGGTCTTTGTATGGAACCTAAT**TTTTTTGTACTGCAAAAAA**GTACATCC
CCGATAAGGCGCC

GTAGACTATGCTAGTATAATAGTCTAC-DSc

TTTTTGCAGTGCAAAAA-DSl **TTTTTTGTACTGCAAAAAA-DSr**

---

37. Thysanoplusia orichalcea NPV isolate P2
130790-130990
JX467702

CACGCTTACGTGCTAGGCGATATTG**AGCTTTTTTGCATTGCAAAAAAGT**TGATTATGTGGTA
GTATATTAGGAGCGTATTGTACAGT**GTAGACTATTCTAATAAAATAGTCTAC**GATTTGTAGA
GTTTGTACTGTATATAGGGTGCCAGCCAAAATTAAAC**TTTTTTGAATTGCAAAAAA**ATTCATT
CTCAATTGTTATTC

GTAGACTATTCTAATAAAATAGTCTAC-DSC

AGCTTTTTTGCATTGCAAAAAAGT –DS1 **TTTTTTGAATTGCAAAAAA-DS2**

---

38. Trichoplusia ni single nucleopolyhedrovirus, complete genome
5833-6033
NC_007383

TTATAAATTCGTTATCATCGAGGTCAA**CTTTTTTGTTGTGCAAAAAAG**GACTCAAAAATGTA
GTATATTGACAGCATATTGTTCAGT**GTAGACTATTCTAGTTAAATAGTCTAC**GATTAGTAGT
TTTTGT***ACTGT***ATATTGATGCCGCCCCGATAACGAAC**TTTTTTGCACTACAAAAAA**`AGACGA
TAATACAATCTAATC

GTAGACTATTCTAGTTAAATAGTCTAC-DSc

CTTTTTTGTTGTGCAAAAAAG-DSl **TTTTTTGCACTACAAAAAA-DSr**
