# Supplementary material for: Identification of a Conserved Non-Protein-Coding Genomic Element that Plays an Essential Role in Alphabaculovirus Pathogenesis
Source: PLoS One. 2014 Apr 16;9(4):e95322. doi: 10.1371/journal.pone.0095322 (PMC3989284; doi:10.1371/journal.pone.0095322)
Supplement: Table S1 — Identity scores between 38 alphabaculovirus CNEs. (PDF) [file pone.0095322.s006.pdf]

Table S1

SeqA	Name	Length	SeqB	Name	Length	Score
1	Adho	156	2	Ador	156	93.0
1	Adho	156	3	Agip	155	75.0
1	Adho	156	4	Agse	155	78.0
1	Adho	156	5	Anpe	156	71.0
1	Adho	156	6	Ag	157	71.0
1	Adho	156	7	Ac	156	77.0
1	Adho	156	8	Apci	155	80.0
1	Adho	156	9	Boma	156	80.0
1	Adho	156	10	Bm	156	80.0
1	Adho	156	11	Cf	155	76.0
1	Adho	156	12	CfDEF	157	72.0
1	Adho	156	13	Cc	156	80.0
1	Adho	156	14	Clbi	155	74.0
1	Adho	156	15	Ecob	156	74.0
1	Adho	156	16	Eppo	156	75.0
1	Adho	156	17	Eups	155	72.0
1	Adho	156	18	HearM	155	78.0
1	Adho	156	19	HearS	155	74.0
1	Adho	156	20	Hycu	156	73.0
1	Adho	156	21	Lese	156	74.0
1	Adho	156	22	Ld	154	74.0
1	Adho	156	23	Lyxy	154	74.0
1	Adho	156	24	Mane	155	69.0
1	Adho	156	25	Mb	155	78.0
1	Adho	156	26	MacoB	155	78.0
1	Adho	156	27	MacoA	155	76.0
1	Adho	156	28	Mavi	156	77.0
1	Adho	156	29	Orle	156	78.0
1	Adho	156	30	Op	156	75.0
1	Adho	156	31	Plxy	156	77.0
1	Adho	156	32	Ro	156	74.0
1	Adho	156	33	Se	155	78.0
1	Adho	156	34	Sf	155	78.0
1	Adho	156	35	SpltII	155	77.0
1	Adho	156	36	SpltG2	154	67.0
1	Adho	156	37	Thor	156	78.0
1	Adho	156	38	Tn	156	73.0
2	Ador	156	3	Agip	155	77.0
2	Ador	156	4	Agse	155	76.0
2	Ador	156	5	Anpe	156	70.0
2	Ador	156	6	Ag	157	70.0
2	Ador	156	7	Ac	156	78.0
2	Ador	156	8	Apci	155	79.0
2	Ador	156	9	Boma	156	80.0
2	Ador	156	10	Bm	156	80.0
2	Ador	156	11	Cf	155	75.0
2	Ador	156	12	CfDEF	157	69.0
2	Ador	156	13	Cc	156	78.0
2	Ador	156	14	Clbi	155	77.0
2	Ador	156	15	Ecob	156	73.0
2	Ador	156	16	Eppo	156	76.0
2	Ador	156	17	Eups	155	75.0
2	Ador	156	18	HearM	155	76.0
2	Ador	156	19	HearS	155	73.0
2	Ador	156	20	Hycu	156	73.0
2	Ador	156	21	Lese	156	72.0
2	Ador	156	22	Ld	154	69.0
2	Ador	156	23	Lyxy	154	74.0
2	Ador	156	24	Mane	155	69.0
2	Ador	156	25	Mb	155	75.0
2	Ador	156	26	MacoB	155	76.0
2	Ador	156	27	MacoA	155	78.0
2	Ador	156	28	Mavi	156	76.0
2	Ador	156	29	Orle	156	75.0
2	Ador	156	30	Op	156	73.0
2	Ador	156	31	Plxy	156	78.0
2	Ador	156	32	Ro	156	77.0
2	Ador	156	33	Se	155	77.0
2	Ador	156	34	Sf	155	78.0
2	Ador	156	35	SpltII	155	74.0
2	Ador	156	36	SpltG2	154	70.0
2	Ador	156	37	Thor	156	77.0
2	Ador	156	38	Tn	156	75.0
3	Agip	155	4	Agse	155	85.0
3	Agip	155	5	Anpe	156	72.0
3	Agip	155	6	Ag	157	74.0

3	Agip	155	7	Ac	156	78.0
3	Agip	155	8	Apci	155	81.0
3	Agip	155	9	Boma	156	74.0
3	Agip	155	10	Bm	156	74.0
3	Agip	155	11	Cf	155	81.0
3	Agip	155	12	CfDEF	157	71.0
3	Agip	155	13	Cc	156	79.0
3	Agip	155	14	Clbi	155	74.0
3	Agip	155	15	Ecob	156	77.0
3	Agip	155	16	Eppo	156	74.0
3	Agip	155	17	Eups	155	78.0
3	Agip	155	18	HearM	155	83.0
3	Agip	155	19	HearS	155	81.0
3	Agip	155	20	Hycu	156	72.0
3	Agip	155	21	Lese	156	79.0
3	Agip	155	22	Ld	154	72.0
3	Agip	155	23	Lyxy	154	77.0
3	Agip	155	24	Mane	155	77.0
3	Agip	155	25	Mb	155	81.0
3	Agip	155	26	MacoB	155	81.0
3	Agip	155	27	MacoA	155	82.0
3	Agip	155	28	Mavi	156	75.0
3	Agip	155	29	Orle	156	80.0
3	Agip	155	30	Op	156	71.0
3	Agip	155	31	Plxy	156	78.0
3	Agip	155	32	Ro	156	78.0
3	Agip	155	33	Se	155	89.0
3	Agip	155	34	Sf	155	90.0
3	Agip	155	35	SpltII	155	88.0
3	Agip	155	36	SpltG2	154	77.0
3	Agip	155	37	Thor	156	73.0
3	Agip	155	38	Tn	156	79.0
4	Agse	155	5	Anpe	156	76.0
4	Agse	155	6	Ag	157	75.0
4	Agse	155	7	Ac	156	75.0
4	Agse	155	8	Apci	155	81.0
4	Agse	155	9	Boma	156	76.0
4	Agse	155	10	Bm	156	76.0
4	Agse	155	11	Cf	155	81.0
4	Agse	155	12	CfDEF	157	74.0
4	Agse	155	13	Cc	156	78.0
4	Agse	155	14	Clbi	155	76.0
4	Agse	155	15	Ecob	156	76.0
4	Agse	155	16	Eppo	156	77.0
4	Agse	155	17	Eups	155	81.0
4	Agse	155	18	HearM	155	86.0
4	Agse	155	19	HearS	155	72.0
4	Agse	155	20	Hycu	156	75.0
4	Agse	155	21	Lese	156	78.0
4	Agse	155	22	Ld	154	74.0
4	Agse	155	23	Lyxy	154	79.0
4	Agse	155	24	Mane	155	77.0
4	Agse	155	25	Mb	155	85.0
4	Agse	155	26	MacoB	155	85.0
4	Agse	155	27	MacoA	155	85.0
4	Agse	155	28	Mavi	156	75.0
4	Agse	155	29	Orle	156	81.0
4	Agse	155	30	Op	156	74.0
4	Agse	155	31	Plxy	156	75.0
4	Agse	155	32	Ro	156	77.0
4	Agse	155	33	Se	155	87.0
4	Agse	155	34	Sf	155	83.0
4	Agse	155	35	SpltII	155	85.0
4	Agse	155	36	SpltG2	154	75.0
4	Agse	155	37	Thor	156	75.0
4	Agse	155	38	Tn	156	78.0
5	Anpe	156	6	Ag	157	79.0
5	Anpe	156	7	Ac	156	80.0
5	Anpe	156	8	Apci	155	70.0
5	Anpe	156	9	Boma	156	77.0
5	Anpe	156	10	Bm	156	77.0
5	Anpe	156	11	Cf	155	80.0
5	Anpe	156	12	CfDEF	157	82.0
5	Anpe	156	13	Cc	156	76.0
5	Anpe	156	14	Clbi	155	70.0
5	Anpe	156	15	Ecob	156	73.0
5	Anpe	156	16	Eppo	156	77.0
5	Anpe	156	17	Eups	155	69.0
5	Anpe	156	18	HearM	155	77.0
5	Anpe	156	19	HearS	155	75.0

5	Anpe	156	20	Hycu	156	85.0
5	Anpe	156	21	Lese	156	73.0
5	Anpe	156	22	Ld	154	69.0
5	Anpe	156	23	Lyxy	154	68.0
5	Anpe	156	24	Mane	155	76.0
5	Anpe	156	25	Mb	155	77.0
5	Anpe	156	26	MacoB	155	76.0
5	Anpe	156	27	MacoA	155	74.0
5	Anpe	156	28	Mavi	156	83.0
5	Anpe	156	29	Orle	156	78.0
5	Anpe	156	30	Op	156	85.0
5	Anpe	156	31	Plxy	156	80.0
5	Anpe	156	32	Ro	156	80.0
5	Anpe	156	33	Se	155	74.0
5	Anpe	156	34	Sf	155	76.0
5	Anpe	156	35	SpltII	155	74.0
5	Anpe	156	36	SpltG2	154	70.0
5	Anpe	156	37	Thor	156	80.0
5	Anpe	156	38	Tn	156	74.0
6	Ag	157	7	Ac	156	83.0
6	Ag	157	8	Apci	155	71.0
6	Ag	157	9	Boma	156	79.0
6	Ag	157	10	Bm	156	79.0
6	Ag	157	11	Cf	155	80.0
6	Ag	157	12	CfDEF	157	92.0
6	Ag	157	13	Cc	156	76.0
6	Ag	157	14	Clbi	155	71.0
6	Ag	157	15	Ecob	156	69.0
6	Ag	157	16	Eppo	156	83.0
6	Ag	157	17	Eups	155	70.0
6	Ag	157	18	HearM	155	77.0
6	Ag	157	19	HearS	155	73.0
6	Ag	157	20	Hycu	156	82.0
6	Ag	157	21	Lese	156	71.0
6	Ag	157	22	Ld	154	72.0
6	Ag	157	23	Lyxy	154	73.0
6	Ag	157	24	Mane	155	72.0
6	Ag	157	25	Mb	155	77.0
6	Ag	157	26	MacoB	155	78.0
6	Ag	157	27	MacoA	155	79.0
6	Ag	157	28	Mavi	156	80.0
6	Ag	157	29	Orle	156	75.0
6	Ag	157	30	Op	156	83.0
6	Ag	157	31	Plxy	156	83.0
6	Ag	157	32	Ro	156	80.0
6	Ag	157	33	Se	155	73.0
6	Ag	157	34	Sf	155	78.0
6	Ag	157	35	SpltII	155	74.0
6	Ag	157	36	SpltG2	154	72.0
6	Ag	157	37	Thor	156	80.0
6	Ag	157	38	Tn	156	72.0
7	Ac	156	8	Apci	155	78.0
7	Ac	156	9	Boma	156	91.0
7	Ac	156	10	Bm	156	91.0
7	Ac	156	11	Cf	155	73.0
7	Ac	156	12	CfDEF	157	80.0
7	Ac	156	13	Cc	156	76.0
7	Ac	156	14	Clbi	155	81.0
7	Ac	156	15	Ecob	156	73.0
7	Ac	156	16	Eppo	156	80.0
7	Ac	156	17	Eups	155	72.0
7	Ac	156	18	HearM	155	83.0
7	Ac	156	19	HearS	155	76.0
7	Ac	156	20	Hycu	156	78.0
7	Ac	156	21	Lese	156	69.0
7	Ac	156	22	Ld	154	72.0
7	Ac	156	23	Lyxy	154	72.0
7	Ac	156	24	Mane	155	76.0
7	Ac	156	25	Mb	155	81.0
7	Ac	156	26	MacoB	155	81.0
7	Ac	156	27	MacoA	155	81.0
7	Ac	156	28	Mavi	156	91.0
7	Ac	156	29	Orle	156	78.0
7	Ac	156	30	Op	156	80.0
7	Ac	156	31	Plxy	156	100.0
7	Ac	156	32	Ro	156	94.0
7	Ac	156	33	Se	155	76.0
7	Ac	156	34	Sf	155	77.0
7	Ac	156	35	SpltII	155	76.0
7	Ac	156	36	SpltG2	154	74.0

7	Ac	156	37	Thor	156	89.0
7	Ac	156	38	Tn	156	78.0
8	Apci	155	9	Boma	156	75.0
8	Apci	155	10	Bm	156	75.0
8	Apci	155	11	Cf	155	76.0
8	Apci	155	12	CfDEF	157	72.0
8	Apci	155	13	Cc	156	75.0
8	Apci	155	14	Clbi	155	75.0
8	Apci	155	15	Ecob	156	80.0
8	Apci	155	16	Eppo	156	72.0
8	Apci	155	17	Eups	155	71.0
8	Apci	155	18	HearM	155	78.0
8	Apci	155	19	HearS	155	78.0
8	Apci	155	20	Hycu	156	72.0
8	Apci	155	21	Lese	156	75.0
8	Apci	155	22	Ld	154	72.0
8	Apci	155	23	Lyxy	154	75.0
8	Apci	155	24	Mane	155	75.0
8	Apci	155	25	Mb	155	76.0
8	Apci	155	26	MacoB	155	76.0
8	Apci	155	27	MacoA	155	78.0
8	Apci	155	28	Mavi	156	74.0
8	Apci	155	29	Orle	156	78.0
8	Apci	155	30	Op	156	74.0
8	Apci	155	31	Plxy	156	77.0
8	Apci	155	32	Ro	156	74.0
8	Apci	155	33	Se	155	80.0
8	Apci	155	34	Sf	155	77.0
8	Apci	155	35	SpltII	155	81.0
8	Apci	155	36	SpltG2	154	74.0
8	Apci	155	37	Thor	156	78.0
8	Apci	155	38	Tn	156	75.0
9	Boma	156	10	Bm	156	100.0
9	Boma	156	11	Cf	155	71.0
9	Boma	156	12	CfDEF	157	79.0
9	Boma	156	13	Cc	156	81.0
9	Boma	156	14	Clbi	155	77.0
9	Boma	156	15	Ecob	156	73.0
9	Boma	156	16	Eppo	156	78.0
9	Boma	156	17	Eups	155	76.0
9	Boma	156	18	HearM	155	80.0
9	Boma	156	19	HearS	155	80.0
9	Boma	156	20	Hycu	156	75.0
9	Boma	156	21	Lese	156	69.0
9	Boma	156	22	Ld	154	74.0
9	Boma	156	23	Lyxy	154	73.0
9	Boma	156	24	Mane	155	73.0
9	Boma	156	25	Mb	155	76.0
9	Boma	156	26	MacoB	155	76.0
9	Boma	156	27	MacoA	155	78.0
9	Boma	156	28	Mavi	156	85.0
9	Boma	156	29	Orle	156	80.0
9	Boma	156	30	Op	156	75.0
9	Boma	156	31	Plxy	156	91.0
9	Boma	156	32	Ro	156	91.0
9	Boma	156	33	Se	155	74.0
9	Boma	156	34	Sf	155	76.0
9	Boma	156	35	SpltII	155	76.0
9	Boma	156	36	SpltG2	154	76.0
9	Boma	156	37	Thor	156	85.0
9	Boma	156	38	Tn	156	77.0
10	Bm	156	11	Cf	155	71.0
10	Bm	156	12	CfDEF	157	79.0
10	Bm	156	13	Cc	156	81.0
10	Bm	156	14	Clbi	155	77.0
10	Bm	156	15	Ecob	156	73.0
10	Bm	156	16	Eppo	156	78.0
10	Bm	156	17	Eups	155	76.0
10	Bm	156	18	HearM	155	80.0
10	Bm	156	19	HearS	155	80.0
10	Bm	156	20	Hycu	156	75.0
10	Bm	156	21	Lese	156	69.0
10	Bm	156	22	Ld	154	74.0
10	Bm	156	23	Lyxy	154	73.0
10	Bm	156	24	Mane	155	73.0
10	Bm	156	25	Mb	155	76.0
10	Bm	156	26	MacoB	155	76.0
10	Bm	156	27	MacoA	155	78.0
10	Bm	156	28	Mavi	156	85.0
10	Bm	156	29	Orle	156	80.0

10	Bm	156	30	Op	156	75.0
10	Bm	156	31	Plxy	156	91.0
10	Bm	156	32	Ro	156	91.0
10	Bm	156	33	Se	155	74.0
10	Bm	156	34	Sf	155	76.0
10	Bm	156	35	SpltII	155	76.0
10	Bm	156	36	SpltG2	154	76.0
10	Bm	156	37	Thor	156	85.0
10	Bm	156	38	Tn	156	77.0
11	Cf	155	12	CfDEF	157	81.0
11	Cf	155	13	Cc	156	77.0
11	Cf	155	14	Clbi	155	74.0
11	Cf	155	15	Ecob	156	74.0
11	Cf	155	16	Eppo	156	78.0
11	Cf	155	17	Eups	155	74.0
11	Cf	155	18	HearM	155	74.0
11	Cf	155	19	HearS	155	73.0
11	Cf	155	20	Hycu	156	82.0
11	Cf	155	21	Lese	156	73.0
11	Cf	155	22	Ld	154	71.0
11	Cf	155	23	Lyxy	154	75.0
11	Cf	155	24	Mane	155	76.0
11	Cf	155	25	Mb	155	74.0
11	Cf	155	26	MacoB	155	74.0
11	Cf	155	27	MacoA	155	75.0
11	Cf	155	28	Mavi	156	78.0
11	Cf	155	29	Orle	156	80.0
11	Cf	155	30	Op	156	80.0
11	Cf	155	31	Plxy	156	72.0
11	Cf	155	32	Ro	156	73.0
11	Cf	155	33	Se	155	80.0
11	Cf	155	34	Sf	155	82.0
11	Cf	155	35	SpltII	155	79.0
11	Cf	155	36	SpltG2	154	74.0
11	Cf	155	37	Thor	156	78.0
11	Cf	155	38	Tn	156	74.0
12	CfDEF	157	13	Cc	156	75.0
12	CfDEF	157	14	Clbi	155	73.0
12	CfDEF	157	15	Ecob	156	68.0
12	CfDEF	157	16	Eppo	156	84.0
12	CfDEF	157	17	Eups	155	69.0
12	CfDEF	157	18	HearM	155	72.0
12	CfDEF	157	19	HearS	155	72.0
12	CfDEF	157	20	Hycu	156	83.0
12	CfDEF	157	21	Lese	156	71.0
12	CfDEF	157	22	Ld	154	70.0
12	CfDEF	157	23	Lyxy	154	73.0
12	CfDEF	157	24	Mane	155	76.0
12	CfDEF	157	25	Mb	155	72.0
12	CfDEF	157	26	MacoB	155	72.0
12	CfDEF	157	27	MacoA	155	78.0
12	CfDEF	157	28	Mavi	156	79.0
12	CfDEF	157	29	Orle	156	75.0
12	CfDEF	157	30	Op	156	83.0
12	CfDEF	157	31	Plxy	156	80.0
12	CfDEF	157	32	Ro	156	83.0
12	CfDEF	157	33	Se	155	71.0
12	CfDEF	157	34	Sf	155	78.0
12	CfDEF	157	35	SpltII	155	70.0
12	CfDEF	157	36	SpltG2	154	71.0
12	CfDEF	157	37	Thor	156	80.0
12	CfDEF	157	38	Tn	156	72.0
13	Cc	156	14	Clbi	155	76.0
13	Cc	156	15	Ecob	156	76.0
13	Cc	156	16	Eppo	156	77.0
13	Cc	156	17	Eups	155	78.0
13	Cc	156	18	HearM	155	79.0
13	Cc	156	19	HearS	155	83.0
13	Cc	156	20	Hycu	156	78.0
13	Cc	156	21	Lese	156	71.0
13	Cc	156	22	Ld	154	74.0
13	Cc	156	23	Lyxy	154	74.0
13	Cc	156	24	Mane	155	74.0
13	Cc	156	25	Mb	155	79.0
13	Cc	156	26	MacoB	155	78.0
13	Cc	156	27	MacoA	155	80.0
13	Cc	156	28	Mavi	156	80.0
13	Cc	156	29	Orle	156	81.0
13	Cc	156	30	Op	156	75.0
13	Cc	156	31	Plxy	156	76.0

13	Cc	156	32	Ro	156	81.0
13	Cc	156	33	Se	155	80.0
13	Cc	156	34	Sf	155	81.0
13	Cc	156	35	SpltII	155	78.0
13	Cc	156	36	SpltG2	154	79.0
13	Cc	156	37	Thor	156	80.0
13	Cc	156	38	Tn	156	90.0
14	Clbi	155	15	Ecob	156	72.0
14	Clbi	155	16	Eppo	156	72.0
14	Clbi	155	17	Eups	155	78.0
14	Clbi	155	18	HearM	155	81.0
14	Clbi	155	19	HearS	155	77.0
14	Clbi	155	20	Hycu	156	69.0
14	Clbi	155	21	Lese	156	69.0
14	Clbi	155	22	Ld	154	68.0
14	Clbi	155	23	Lyxy	154	74.0
14	Clbi	155	24	Mane	155	69.0
14	Clbi	155	25	Mb	155	80.0
14	Clbi	155	26	MacoB	155	81.0
14	Clbi	155	27	MacoA	155	79.0
14	Clbi	155	28	Mavi	156	76.0
14	Clbi	155	29	Orle	156	76.0
14	Clbi	155	30	Op	156	69.0
14	Clbi	155	31	Plxy	156	81.0
14	Clbi	155	32	Ro	156	79.0
14	Clbi	155	33	Se	155	79.0
14	Clbi	155	34	Sf	155	79.0
14	Clbi	155	35	SpltII	155	74.0
14	Clbi	155	36	SpltG2	154	75.0
14	Clbi	155	37	Thor	156	74.0
14	Clbi	155	38	Tn	156	73.0
15	Ecob	156	16	Eppo	156	75.0
15	Ecob	156	17	Eups	155	71.0
15	Ecob	156	18	HearM	155	73.0
15	Ecob	156	19	HearS	155	78.0
15	Ecob	156	20	Hycu	156	75.0
15	Ecob	156	21	Lese	156	73.0
15	Ecob	156	22	Ld	154	71.0
15	Ecob	156	23	Lyxy	154	71.0
15	Ecob	156	24	Mane	155	70.0
15	Ecob	156	25	Mb	155	72.0
15	Ecob	156	26	MacoB	155	72.0
15	Ecob	156	27	MacoA	155	77.0
15	Ecob	156	28	Mavi	156	72.0
15	Ecob	156	29	Orle	156	79.0
15	Ecob	156	30	Op	156	70.0
15	Ecob	156	31	Plxy	156	73.0
15	Ecob	156	32	Ro	156	71.0
15	Ecob	156	33	Se	155	77.0
15	Ecob	156	34	Sf	155	76.0
15	Ecob	156	35	SpltII	155	80.0
15	Ecob	156	36	SpltG2	154	77.0
15	Ecob	156	37	Thor	156	75.0
15	Ecob	156	38	Tn	156	78.0
16	Eppo	156	17	Eups	155	77.0
16	Eppo	156	18	HearM	155	78.0
16	Eppo	156	19	HearS	155	72.0
16	Eppo	156	20	Hycu	156	81.0
16	Eppo	156	21	Lese	156	70.0
16	Eppo	156	22	Ld	154	71.0
16	Eppo	156	23	Lyxy	154	72.0
16	Eppo	156	24	Mane	155	71.0
16	Eppo	156	25	Mb	155	76.0
16	Eppo	156	26	MacoB	155	77.0
16	Eppo	156	27	MacoA	155	74.0
16	Eppo	156	28	Mavi	156	78.0
16	Eppo	156	29	Orle	156	72.0
16	Eppo	156	30	Op	156	81.0
16	Eppo	156	31	Plxy	156	80.0
16	Eppo	156	32	Ro	156	79.0
16	Eppo	156	33	Se	155	73.0
16	Eppo	156	34	Sf	155	73.0
16	Eppo	156	35	SpltII	155	72.0
16	Eppo	156	36	SpltG2	154	70.0
16	Eppo	156	37	Thor	156	80.0
16	Eppo	156	38	Tn	156	75.0
17	Eups	155	18	HearM	155	73.0
17	Eups	155	19	HearS	155	76.0
17	Eups	155	20	Hycu	156	72.0
17	Eups	155	21	Lese	156	68.0

17	Eups	155	22	Ld	154	70.0
17	Eups	155	23	Lyxy	154	75.0
17	Eups	155	24	Mane	155	68.0
17	Eups	155	25	Mb	155	72.0
17	Eups	155	26	MacoB	155	72.0
17	Eups	155	27	MacoA	155	74.0
17	Eups	155	28	Mavi	156	71.0
17	Eups	155	29	Orle	156	79.0
17	Eups	155	30	Op	156	67.0
17	Eups	155	31	Plxy	156	72.0
17	Eups	155	32	Ro	156	72.0
17	Eups	155	33	Se	155	81.0
17	Eups	155	34	Sf	155	76.0
17	Eups	155	35	SpltII	155	77.0
17	Eups	155	36	SpltG2	154	70.0
17	Eups	155	37	Thor	156	72.0
17	Eups	155	38	Tn	156	81.0
18	HearM	155	19	HearS	155	76.0
18	HearM	155	20	Hycu	156	79.0
18	HearM	155	21	Lese	156	75.0
18	HearM	155	22	Ld	154	72.0
18	HearM	155	23	Lyxy	154	79.0
18	HearM	155	24	Mane	155	75.0
18	HearM	155	25	Mb	155	98.0
18	HearM	155	26	MacoB	155	98.0
18	HearM	155	27	MacoA	155	92.0
18	HearM	155	28	Mavi	156	79.0
18	HearM	155	29	Orle	156	78.0
18	HearM	155	30	Op	156	73.0
18	HearM	155	31	Plxy	156	83.0
18	HearM	155	32	Ro	156	78.0
18	HearM	155	33	Se	155	84.0
18	HearM	155	34	Sf	155	80.0
18	HearM	155	35	SpltII	155	83.0
18	HearM	155	36	SpltG2	154	79.0
18	HearM	155	37	Thor	156	77.0
18	HearM	155	38	Tn	156	72.0
19	HearS	155	20	Hycu	156	71.0
19	HearS	155	21	Lese	156	76.0
19	HearS	155	22	Ld	154	68.0
19	HearS	155	23	Lyxy	154	73.0
19	HearS	155	24	Mane	155	77.0
19	HearS	155	25	Mb	155	78.0
19	HearS	155	26	MacoB	155	77.0
19	HearS	155	27	MacoA	155	76.0
19	HearS	155	28	Mavi	156	76.0
19	HearS	155	29	Orle	156	79.0
19	HearS	155	30	Op	156	73.0
19	HearS	155	31	Plxy	156	76.0
19	HearS	155	32	Ro	156	76.0
19	HearS	155	33	Se	155	76.0
19	HearS	155	34	Sf	155	76.0
19	HearS	155	35	SpltII	155	80.0
19	HearS	155	36	SpltG2	154	76.0
19	HearS	155	37	Thor	156	73.0
19	HearS	155	38	Tn	156	78.0
20	Hycu	156	21	Lese	156	79.0
20	Hycu	156	22	Ld	154	73.0
20	Hycu	156	23	Lyxy	154	72.0
20	Hycu	156	24	Mane	155	71.0
20	Hycu	156	25	Mb	155	78.0
20	Hycu	156	26	MacoB	155	77.0
20	Hycu	156	27	MacoA	155	78.0
20	Hycu	156	28	Mavi	156	83.0
20	Hycu	156	29	Orle	156	78.0
20	Hycu	156	30	Op	156	84.0
20	Hycu	156	31	Plxy	156	78.0
20	Hycu	156	32	Ro	156	76.0
20	Hycu	156	33	Se	155	74.0
20	Hycu	156	34	Sf	155	75.0
20	Hycu	156	35	SpltII	155	75.0
20	Hycu	156	36	SpltG2	154	75.0
20	Hycu	156	37	Thor	156	80.0
20	Hycu	156	38	Tn	156	73.0
21	Lese	156	22	Ld	154	70.0
21	Lese	156	23	Lyxy	154	71.0
21	Lese	156	24	Mane	155	70.0
21	Lese	156	25	Mb	155	76.0
21	Lese	156	26	MacoB	155	76.0
21	Lese	156	27	MacoA	155	80.0

21	Lese	156	28	Mavi	156	75.0
21	Lese	156	29	Orle	156	80.0
21	Lese	156	30	Op	156	72.0
21	Lese	156	31	Plxy	156	69.0
21	Lese	156	32	Ro	156	67.0
21	Lese	156	33	Se	155	74.0
21	Lese	156	34	Sf	155	76.0
21	Lese	156	35	SpltII	155	80.0
21	Lese	156	36	SpltG2	154	74.0
21	Lese	156	37	Thor	156	69.0
21	Lese	156	38	Tn	156	72.0
22	Ld	154	23	Lyxy	154	86.0
22	Ld	154	24	Mane	155	70.0
22	Ld	154	25	Mb	155	73.0
22	Ld	154	26	MacoB	155	72.0
22	Ld	154	27	MacoA	155	72.0
22	Ld	154	28	Mavi	156	72.0
22	Ld	154	29	Orle	156	70.0
22	Ld	154	30	Op	156	70.0
22	Ld	154	31	Plxy	156	72.0
22	Ld	154	32	Ro	156	72.0
22	Ld	154	33	Se	155	76.0
22	Ld	154	34	Sf	155	73.0
22	Ld	154	35	SpltII	155	75.0
22	Ld	154	36	SpltG2	154	72.0
22	Ld	154	37	Thor	156	71.0
22	Ld	154	38	Tn	156	70.0
23	Lyxy	154	24	Mane	155	74.0
23	Lyxy	154	25	Mb	155	77.0
23	Lyxy	154	26	MacoB	155	78.0
23	Lyxy	154	27	MacoA	155	76.0
23	Lyxy	154	28	Mavi	156	68.0
23	Lyxy	154	29	Orle	156	75.0
23	Lyxy	154	30	Op	156	73.0
23	Lyxy	154	31	Plxy	156	73.0
23	Lyxy	154	32	Ro	156	71.0
23	Lyxy	154	33	Se	155	74.0
23	Lyxy	154	34	Sf	155	79.0
23	Lyxy	154	35	SpltII	155	76.0
23	Lyxy	154	36	SpltG2	154	69.0
23	Lyxy	154	37	Thor	156	73.0
23	Lyxy	154	38	Tn	156	74.0
24	Mane	155	25	Mb	155	74.0
24	Mane	155	26	MacoB	155	75.0
24	Mane	155	27	MacoA	155	70.0
24	Mane	155	28	Mavi	156	74.0
24	Mane	155	29	Orle	156	76.0
24	Mane	155	30	Op	156	73.0
24	Mane	155	31	Plxy	156	76.0
24	Mane	155	32	Ro	156	76.0
24	Mane	155	33	Se	155	74.0
24	Mane	155	34	Sf	155	78.0
24	Mane	155	35	SpltII	155	76.0
24	Mane	155	36	SpltG2	154	74.0
24	Mane	155	37	Thor	156	76.0
24	Mane	155	38	Tn	156	72.0
25	Mb	155	26	MacoB	155	98.0
25	Mb	155	27	MacoA	155	92.0
25	Mb	155	28	Mavi	156	79.0
25	Mb	155	29	Orle	156	77.0
25	Mb	155	30	Op	156	68.0
25	Mb	155	31	Plxy	156	80.0
25	Mb	155	32	Ro	156	79.0
25	Mb	155	33	Se	155	83.0
25	Mb	155	34	Sf	155	80.0
25	Mb	155	35	SpltII	155	81.0
25	Mb	155	36	SpltG2	154	78.0
25	Mb	155	37	Thor	156	80.0
25	Mb	155	38	Tn	156	76.0
26	MacoB	155	27	MacoA	155	92.0
26	MacoB	155	28	Mavi	156	78.0
26	MacoB	155	29	Orle	156	78.0
26	MacoB	155	30	Op	156	73.0
26	MacoB	155	31	Plxy	156	81.0
26	MacoB	155	32	Ro	156	81.0
26	MacoB	155	33	Se	155	84.0
26	MacoB	155	34	Sf	155	80.0
26	MacoB	155	35	SpltII	155	81.0
26	MacoB	155	36	SpltG2	154	78.0
26	MacoB	155	37	Thor	156	79.0

26	MacoB	155	38	Tn	156	75.0
27	MacoA	155	28	Mavi	156	78.0
27	MacoA	155	29	Orle	156	79.0
27	MacoA	155	30	Op	156	75.0
27	MacoA	155	31	Plxy	156	81.0
27	MacoA	155	32	Ro	156	77.0
27	MacoA	155	33	Se	155	83.0
27	MacoA	155	34	Sf	155	81.0
27	MacoA	155	35	SpltII	155	85.0
27	MacoA	155	36	SpltG2	154	80.0
27	MacoA	155	37	Thor	156	70.0
27	MacoA	155	38	Tn	156	76.0
28	Mavi	156	29	Orle	156	80.0
28	Mavi	156	30	Op	156	80.0
28	Mavi	156	31	Plxy	156	91.0
28	Mavi	156	32	Ro	156	89.0
28	Mavi	156	33	Se	155	75.0
28	Mavi	156	34	Sf	155	75.0
28	Mavi	156	35	SpltII	155	75.0
28	Mavi	156	36	SpltG2	154	77.0
28	Mavi	156	37	Thor	156	86.0
28	Mavi	156	38	Tn	156	78.0
29	Orle	156	30	Op	156	74.0
29	Orle	156	31	Plxy	156	78.0
29	Orle	156	32	Ro	156	78.0
29	Orle	156	33	Se	155	79.0
29	Orle	156	34	Sf	155	82.0
29	Orle	156	35	SpltII	155	80.0
29	Orle	156	36	SpltG2	154	78.0
29	Orle	156	37	Thor	156	76.0
29	Orle	156	38	Tn	156	80.0
30	Op	156	31	Plxy	156	80.0
30	Op	156	32	Ro	156	80.0
30	Op	156	33	Se	155	73.0
30	Op	156	34	Sf	155	72.0
30	Op	156	35	SpltII	155	73.0
30	Op	156	36	SpltG2	154	70.0
30	Op	156	37	Thor	156	80.0
30	Op	156	38	Tn	156	71.0
31	Plxy	156	32	Ro	156	94.0
31	Plxy	156	33	Se	155	76.0
31	Plxy	156	34	Sf	155	77.0
31	Plxy	156	35	SpltII	155	76.0
31	Plxy	156	36	SpltG2	154	74.0
31	Plxy	156	37	Thor	156	89.0
31	Plxy	156	38	Tn	156	78.0
32	Ro	156	33	Se	155	78.0
32	Ro	156	34	Sf	155	80.0
32	Ro	156	35	SpltII	155	76.0
32	Ro	156	36	SpltG2	154	76.0
32	Ro	156	37	Thor	156	89.0
32	Ro	156	38	Tn	156	78.0
33	Se	155	34	Sf	155	87.0
33	Se	155	35	SpltII	155	90.0
33	Se	155	36	SpltG2	154	77.0
33	Se	155	37	Thor	156	72.0
33	Se	155	38	Tn	156	77.0
34	Sf	155	35	SpltII	155	85.0
34	Sf	155	36	SpltG2	154	81.0
34	Sf	155	37	Thor	156	77.0
34	Sf	155	38	Tn	156	80.0
35	SpltII	155	36	SpltG2	154	78.0
35	SpltII	155	37	Thor	156	74.0
35	SpltII	155	38	Tn	156	76.0
36	SpltG2	154	37	Thor	156	77.0
36	SpltG2	154	38	Tn	156	81.0
37	Thor	156	38	Tn	156	79.0
