# Supplementary material for: Identification of a Conserved Non-Protein-Coding Genomic Element that Plays an Essential Role in Alphabaculovirus Pathogenesis
Source: PLoS One. 2014 Apr 16;9(4):e95322. doi: 10.1371/journal.pone.0095322 (PMC3989284; doi:10.1371/journal.pone.0095322)
Supplement: Table S2 — A comparison of the alphabaculovirus genome AT content and the CNE AT content. (PDF) [file pone.0095322.s007.pdf]

Table S2

N	Virus	Accession	Genomic AT content (%)	CNE AT content (%)	Genomic AT/ CNE AT ratio
1	*Apocheima cinerarium* NPV (ApciNPV)	NC_018504	66.6	67.3	1.01
2	*Adoxophyes orana* NPV (AdorNPV)	NC_011423	65.0	70.8	1.09
3	*Adoxophyes honmai* NPV (AdhoNPV)	NC_004690	64.4	68.2	1.06
4	*Ecotropis obliqua* NPV (EcobNPV)	NC_008586	62.4	68.2	1.09
5	*Clanis bilineata* NPV (ClbiNPV)	NC_008293	62.3	73.9	1.19
6	*Maruca vitrata* MNPV (MaviMNPV)	NC_008725	61.4	68.2	1.11
7	*Helicoverpa armigera* SNPV (HearSNPV)	NC_003094	61.1	69.3	1.13
8	*Chrysodeixis chalcites* NPV (ChchNPV)	NC_007151	61.0	70.1	1.15
9	*Trichoplusia ni* SNPV (TniSNPV)	NC_007383	61.0	68.2	1.12
10	*Rachiplusia ou* MNPV (RoMNPV)	NC_004323	60.9	70.1	1.15
11	*Thysanoplusia orichalcea* MNPV (ThorMNPV)	JX467702	60.5	69.5	1.15
12	*Mamestra brassicae* MNPV (MbMNPV)	JQ_798165	60.1	66.7	1.11
13	*Mamestra configurata* NPV-B (MacoNPV-B)	NC_004117	60.0	66.7	1.11
14	*Helicoverpa armigera* MNPV (HearMNPV)	NC_011615	59.9	66.0	1.10
15	*Bombyx mandarina* NPV (BomaNPV)	NC_012672	59.8	71.4	1.24
16	*Spodoptera frugiperda* MNPV (SfMNPV)	NC_009011	59.8	69.3	1.16
17	*Bombyx mori* NPV (BmNPV)	NC_001962	59.6	71.4	1.20
18	*Orgyia leucostigma* NPV (Orle NPV)	NC_010276	59.4	*69.5*	1.17
19	*Euproctis pseudoconspersa* NPV (EupsNPV)	NC_012639	59.4	71.9	1.21
20	*Plutella xylostella* MNPV (PlxyMNPV)	NC_008349	59.3	68.2	1.15
21	*Epiphyas postvittana* NPV (EppoNPV)	NC_003083	59.3	68.8	1.16
22	*Autographa californica* MNPV (AcMNPV)	NC_001623	*59.3*	68.2	1.15
23	*Mamestra configurata* NPV-A (MacoNPV-A)	NC_003529	58.3	64.7	1.11
24	*Spodoptera litura* MNPV (SpliMNPV)	NC_003102	57.2	69.1	1.21
25	*Spodoptera exigua* MNPV (SeMNPV)	NC_002169	56.2	66.0	1.17
26	*Anticarsia gemmatalis* MNPV (AgMNPV)	NC_008520	55.5	67.6	1.22
27	*Spodoptera litura* NPVII (SpliNPVII)	NC_011616	55.0	66.7	1.21
28	*Hyphantria cunea* NPV (HycuNPV)	NC_007767	54.5	*64.9*	1.19
29	*Agrotis segetum* NPV (AgseNPV)	NC_007921	54.3	67.3	1.24
30	*Choristoneura fumiferana* defective NPV (CfDEFNPV)	NC_005137	54.2	64.5	1.19
31	*Agrotis ipsilon* MNPV (AgipMNPV)	NC_011345	51.4	69.3	1.35
32	*Leucania separata* NPV (LeseNPV)	NC_008348	51.4	66.2	1.29
33	*Choristoneura fumiferana* MNPV (CfMNPV)	NC_004778	49.9	69.9	1.40
34	*Orgyia pseudotsugata* MNPV (OpMNPV)	NC_001875	48.5	61.5	1.27
35	*Lymantria xylina* MNPV (LyxyMNPV)	NC_013953	46.5	62.5	1.34
36	*Antheraea pernyi* MNPV (AnpeMNPV)	NC_008035	46.5	63.6	1.37
37	*Lymantria dispar* MNPV (LdMNPV)	NC_001973	42.5	55.3	1.30
	AVERAGE		57.4	62.6	1.09
